# Termination factor Rho: From the control of pervasive transcription to cell fate determination in *Bacillus subtilis*

**DOI:** 10.1371/journal.pgen.1006909

**Published:** 2017-07-19

**Authors:** Vladimir Bidnenko, Pierre Nicolas, Aleksandra Grylak-Mielnicka, Olivier Delumeau, Sandrine Auger, Anne Aucouturier, Cyprien Guerin, Francis Repoila, Jacek Bardowski, Stéphane Aymerich, Elena Bidnenko

**Affiliations:** 1 Micalis Institute, INRA, AgroParisTech, Université Paris-Saclay, Jouy-en-Josas, France; 2 MaIAGE, INRA, Université Paris-Saclay, Jouy-en-Josas, France; 3 Institute of Biochemistry and Biophysics PAS, Warsaw, Poland; University of Geneva Medical School, SWITZERLAND

## Abstract

In eukaryotes, RNA species originating from pervasive transcription are regulators of various cellular processes, from the expression of individual genes to the control of cellular development and oncogenesis. In prokaryotes, the function of pervasive transcription and its output on cell physiology is still unknown. Most bacteria possess termination factor Rho, which represses pervasive, mostly antisense, transcription. Here, we investigate the biological significance of Rho-controlled transcription in the Gram-positive model bacterium *Bacillus subtilis*. Rho inactivation strongly affected gene expression in *B*. *subtilis*, as assessed by transcriptome and proteome analysis of a *rho*–null mutant during exponential growth in rich medium. Subsequent physiological analyses demonstrated that a considerable part of Rho-controlled transcription is connected to balanced regulation of three mutually exclusive differentiation programs: cell motility, biofilm formation, and sporulation. In the absence of Rho, several up-regulated sense and antisense transcripts affect key structural and regulatory elements of these differentiation programs, thereby suppressing motility and biofilm formation and stimulating sporulation. We dissected how Rho is involved in the activity of the cell fate decision-making network, centered on the master regulator Spo0A. We also revealed a novel regulatory mechanism of Spo0A activation through Rho-dependent intragenic transcription termination of the protein kinase *kinB* gene. Altogether, our findings indicate that distinct Rho-controlled transcripts are functional and constitute a previously unknown built-in module for the control of cell differentiation in *B*. *subtilis*. In a broader context, our results highlight the recruitment of the termination factor Rho, for which the conserved biological role is probably to repress pervasive transcription, in highly integrated, bacterium-specific, regulatory networks.

## Author summary

*Bacillus subtilis* is a widely used model to study cell differentiation in the bacterial world. This soil-dwelling bacterium can engage in several alternative developmental programs, which generate distinct cell types adapted to different lifestyles, to cope with its complex and changing natural environment. The underlying differentiation control mechanisms are interconnected and tightly regulated, because these physiological and morphological cellular states are mutually exclusive and a correct choice is crucial. Here, we describe a previously unrecognized mechanism that regulates cell fate decisions in *B*. *subtilis*. It is based on the elements of pervasive genome-wide transcription controlled by the termination factor Rho. Pervasive transcription originating from non-defined or cryptic signals is spread throughout bacterial transcriptomes, but its physiological role is not yet well understood. We show that the elements of the Rho-controlled transcriptome affect specific developmental programs in *B*. *subtilis*: cell motility, biofilm formation, and sporulation, by directly or indirectly targeting expression of the key factors of cellular differentiation. Our results illuminate how Rho plays a prominent role in complex and organism-specific regulatory networks by controlling pervasive transcription. These findings rank Rho among the global transcriptional regulators of *B*. *subtilis* and invite systematic exploration of its role in other microorganisms.

## Introduction

Transcription provides the basis for cellular development and metabolism in all living organisms by allowing the expression of the information stored in the DNA sequence of the genes. A different type of transcription not associated with classical, clearly delineated, expression units was discovered nearly fifteen years ago [1]. The term “pervasive transcription” was coined for this non-canonical type of transcription, found in all kingdoms of life, because of its generally genome-wide distribution, initiation from unexpected, often non-defined, or cryptic signals, or its arising from transcriptional read-through at weak or factor-dependent terminators [2–6].

From its discovery, the phenomenon of pervasive transcription raised questions concerning the biological functions of the associated RNA species. Indeed, this potentially futile process could have deleterious effects on cell physiology by interfering with sense transcription or chromosome replication or by compromising genome stability or cellular energy status [5–7]. However, turning it completely off may be difficult and counterproductive from an evolutionary stand-point, since mutations can continuously create new transcription initiation sites or alter the termination of existing transcription units. Indeed, this process may provide raw material for the evolution of novel functional biomolecules [8]. Pervasive transcription may thus be the result of a tradeoff between evolutionary forces and the production of essentially nonfunctional transcripts that are neutral or even slightly deleterious in terms of organism fitness. However, extensive studies in eukaryotes have established pervasive transcription as a fundamental component of the regulatory circuits that notably increases the complexity of gene control [7, 9–11]. The produced non-coding RNAs (ncRNAs) are involved in a wide range of cellular processes, playing crucial roles in development, aging, disease, and the evolution of complex organisms [7, 12, 13].

Pervasive transcription has been found in various bacterial transcriptomes [14–21], but its physiological role is still unclear. At the same time, mechanisms preventing pervasive transcription in bacteria are well known [6]. The transcription termination factor Rho, an ATP-dependent RNA helicase-translocase responsible for the main factor-dependent termination pathway in bacteria, plays an important role in preventing pervasive transcription [22–27]. In contrast to intrinsic terminators, the sequence features required for the function of Rho are complex and poorly defined [23, 24]. Rho is nearly ubiquitous in bacterial genomes and the basic principles of Rho-dependent-termination are conserved across species, despite some structural differences between Rho proteins. Over the past decade, the importance of Rho in gene regulation and its conserved role in the enforcement of transcription-translation coupling, by interrupting transcription of untranslated mRNAs, has been substantiated by studies performed in several bacterial species [6, 25–27]. The major role of Rho in the suppression of pervasive, primarily antisense, transcription has been demonstrated for the Gram-negative and Gram-positive microorganisms *Escherichia coli*, *Bacillus subtilis*, *Staphylococcus aureus*, and *Mycobacterium tuberculosis*, under conditions of Rho depletion [17, 18, 20, 21]. Complete or even partial inactivation of Rho in these bacterial species causes widespread transcription originating from cryptic promoters and read-through of transcription terminators [17, 18, 20, 21]. The biological relevance of this Rho-controlled component of the bacterial transcriptome is poorly understood. In *E*. *coli*, Rho inactivation is lethal, and a single amino acid substitution can cause changes in sense transcript levels and altered cellular fitness in the presence of various nutrients [28]. However, the increase of antisense transcription, due to partial inhibition of Rho, was reported to not interfere with sense transcription or gene expression in *E*. *coli* [18]. Similarly, no correlation between the levels of sense and antisense transcripts has been detected in *M*. *tuberculosis*. However, Rho inactivation in this bacterium significantly affected gene expression and caused cell death in cultures and during infection [21]. In contrast, a negative relationship between sense and antisense transcripts was observed in an *S*. *aureus rho* mutant [20], suggesting that Rho-controlled asRNAs may influence gene expression. Nonetheless, the lack of Rho did not significantly modify the growth behavior of either *B*. *subtilis* or *S*. *aureus* cells under the growth conditions tested [17, 20].

An increasing number of reports support a role of individual ncRNAs and antisense RNAs (asRNAs) in the regulation of gene expression in bacteria [5, 16, 29–33], but it is still accepted that most pervasive transcription represents non-functional and relatively low-level transcriptional noise [18, 34]. However, noise, or random fluctuations in gene expression due to the stochastic character of cellular processes involving low copy number cellular components (e.g. transcription factors or mRNAs) [35, 36], is also an important component of the fundamental processes of development and cell fate decision making in living organisms, from bacteria to mammals, as well as viruses [35–37]. Nonetheless, the influence of pervasive transcription on the regulation of developmental programs has not been experimentally addressed in bacteria.

The Gram-positive, soil dwelling, bacterium *B*. *subtilis* is a model for studying phenotypic heterogeneity and transitional developmental programs in prokaryotes, as it can express distinct cell types associated with specific phenotypes: motility, production of lipopeptide surfactin, genetic competence, biofilm formation, protease production, and sporulation [37–40]. During exponential growth, one sub-population of *B*. *subtilis* cells can synthesize flagella and grow as individual motile cells. The motile state of *B*. *subtilis* populations is determined by the alternative sigma factor, SigD, which drives the expression of genes essential for the synthesis and regulation of the flagellar apparatus [40, 41]. The transition from motility to an alternative type of cellular growth within surface-associated communities, known as biofilms, involves the repression of flagellar genes and activation of genes essential for production of biofilm extracellular matrix composed of polysaccharides, protein fibers and nucleic acids [41–43]. Under conditions of limiting nutrients, a sub-population of *B*. *subtilis* cells can initiate a multistage differentiation program to form highly resistant endospores (spores) [44, 45]. The respective gene networks controlling these mutually exclusive developmental programs are interconnected and share common regulators and regulatory feedback loops, which prevent their simultaneous activation within a cell [41, 43, 44, 46, 47]. The key determinant in the regulation of biofilm formation and sporulation is the master regulator Spo0A, for which the activity depends on its gradually increasing phosphorylation state, determined by a multicomponent phosphorelay system [48–54]. When the concentration of phosphorylated Spo0A (Spo0A~P) is low, biofilm formation and sporulation are repressed by the transcriptional regulator of exponential growth, AbrB, and the biofilm-specific repressor, SinR [55, 56]. This negative control is removed at intermediate levels of Spo0A~P, which activates the biofilm formation program [47, 57–62]. Only cells expressing high levels of Spo0A~P can enter into sporulation [45, 58, 63]. At the same time, matrix production is blocked by high Spo0A~P [43, 56–58]. Thus, the level of Spo0A~P determines heterogeneity of the matrix and spore production in populations of *B*. *subtilis*.

Here, we investigated the impact of pervasive transcription on the physiology of *B*. *subtilis* cells taking advantage of the viability of *B*. *subtilis rho*-null mutant. Comparative transcriptome and proteome analyses of *B*. *subtilis* wild type (WT) and *rho* mutant (RM) strains revealed significant perturbation of the global gene expression landscape in the absence of Rho and highlighted potential alterations of the regulatory networks known to define cell fate in *B*. *subtilis*. Further functional studies demonstrated that at least three of the above-mentioned differentiation programs, motility, biofilm formation, and sporulation were altered in RM cells due to the loss of Rho-mediated control of pervasive transcription. We describe several mechanisms by which Rho directly or indirectly participates in the *in fine* regulation of cell fate decision-making. Rho-controlled transcription represents a new level of regulation of gene expression in the Gram positive bacterium *B*. *subtilis* and the termination factor Rho can be considered among the global transcriptional regulators.

## Results

### Alterations of the transcription landscape in the *B*. *subtilis* RM strain

We reanalyzed the dataset of genome-wide expression profiles previously established for *B*. *subtilis* 168 derivative strain 1012 and its isogenic *rho* mutant (RM) grown exponentially in rich medium as a starting point for dissecting the pathways by which the absence of Rho could affect *B*. *subtilis* physiology [17]. These tiling array data can be visualized on the *B*. *subtilis* expression data browser with expression profiles established for the BSB1 strain, a tryptophan-phototrophic (*trp*+) derivative of 168 (http://genome.jouy.inra.fr/cgi-bin/seb/index.py), [17].

The previous analysis of the RM strain was mainly focused on the detection of transcription outside of the transcribed regions (TRs) detected in the wild type strain (native TRs) [17]. The goal of the present reanalysis was to characterize global changes that affect functional regions (in particular mRNAs) and can lead to phenotypic variations. Therefore, we focused on the differential expression of sense and antisense expression levels aggregated according to a repertoire of 5,875 native TRs including mRNAs and ncRNAs identified in the BSB1 strain (WT) across 104 conditions. In this repertoire, 1,583 transcribed regions outside the Genbank-annotated genes were previously designated as S-segments and are numbered S1-S1583, according to their chromosomal position [17] (S1 Table).

**Fig 1:**
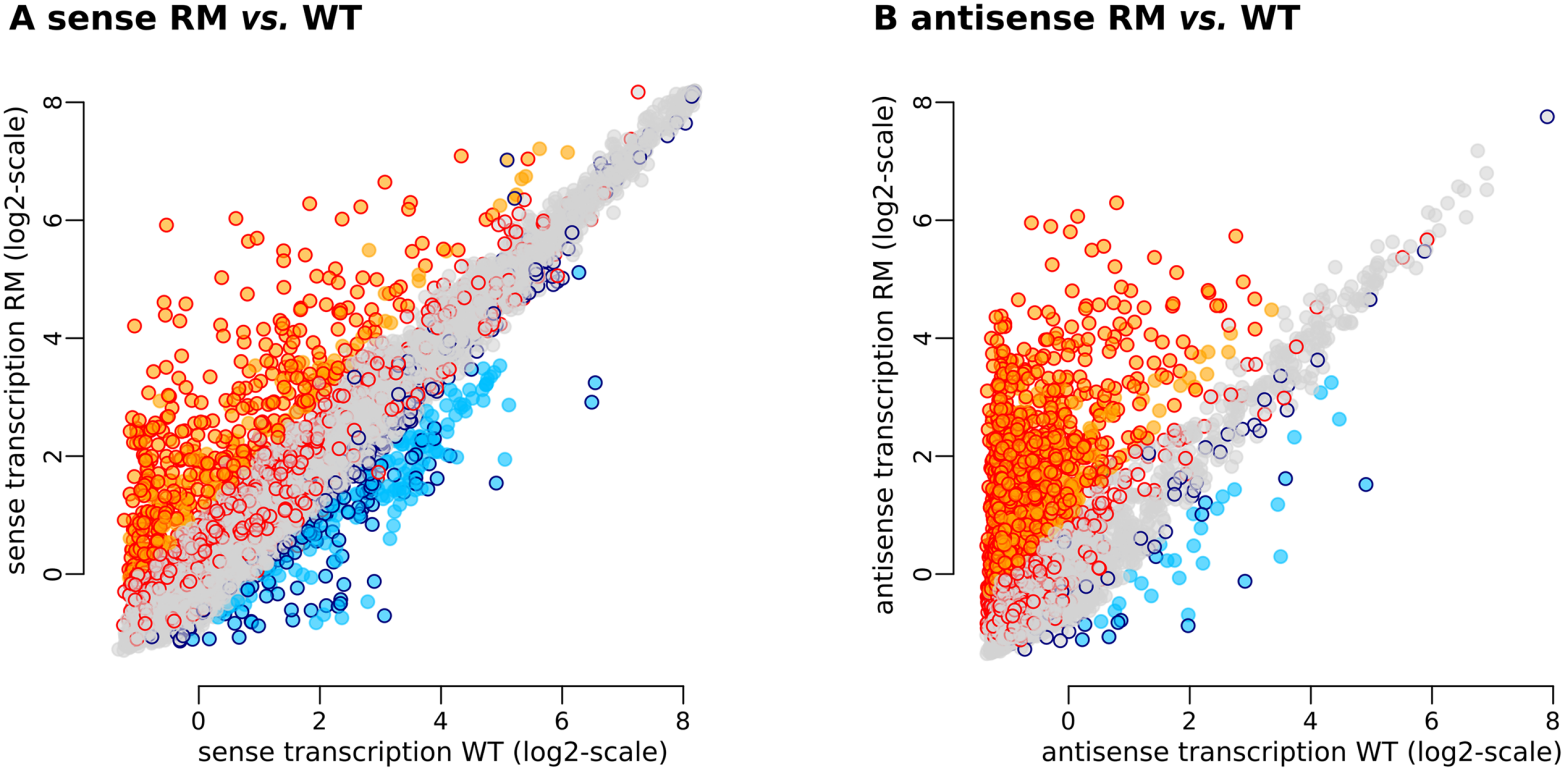
Genome wide effects of *rho* deletion on the *B*. *subtilis* transcriptome during exponential growth in rich medium. (A and B) Transcriptome changes in the sense and antisense strands, respectively. Each point represents one of the 5875 native TRs. Coordinates on x- and y-axes correspond to the normalized expression level (average of three biological replicates) measured with tiling arrays in *B*. *subtilis* 1012 WT and RM, respectively. Background colors of the points indicate TRs whose transcription level is strongly up-regulated (orange) or down-regulated (light blue) in the RM vs. WT comparison made in *B*. *subtilis* 1012 by tiling arrays. Contour colors of the points indicate TRs whose transcription level is strongly up-regulated (red) or down-regulated (dark blue) in the RM vs. WT comparison made in *B*. *subtilis* NCIB 3610 by RNA-Seq.

Significant changes in expression (log2 RM/WT ≥ 1 or ≤ -1) consisted primarily in up-regulation of the antisense strand, in agreement with our initial observations [17]. The detected changes were decomposed into 456 up-regulations and 223 down-regulations on the sense strand (Fig 1A) and 1,446 up-regulations and 36 down-regulations on the antisense strand (Fig 1B). Many of these changes exceeded a four-fold threshold (log2 RM/WT ≥ 2 or ≤ -2): 162 up- and 38 down-regulations on the sense strand, and 613 up- and seven down-regulations on the antisense strand. S1 Table presents the detailed results of this re-analysis.

We used the non-domesticated NCIB 3610 strain for subsequent physiological analysis of *rho* mutants. This strain is a member of the 168-like group of strains originating from the Marburg ancestor and characterized by highly similar genome sequences [64]. Thus we also collected new transcriptome data to investigate the effect of *rho* deletion in the NCIB 3610 background. These new RNA-Seq-based data for the NCIB 3610 WT and NCIB 3610 RM strains (Materials and methods) are consistent with the previous data obtained by tiling array in the *B*. *subtilis* 1012 background. Differential expression analysis of the NCIB 3610 RM strain identified 1,029 up-regulations and 375 down-regulations of the sense strand, along with 2,115 up-regulations and 72 down-regulations of the anti-sense strand (S1 Table). Approximately 80% of the up-regulations identified in 1012 RM were also found in NCIB3610 RM; the correspondence between data sets was lower for the sense strand (≈35%), but still highly statistically significant (Fig 1A and 1B).

**Fig 2:**
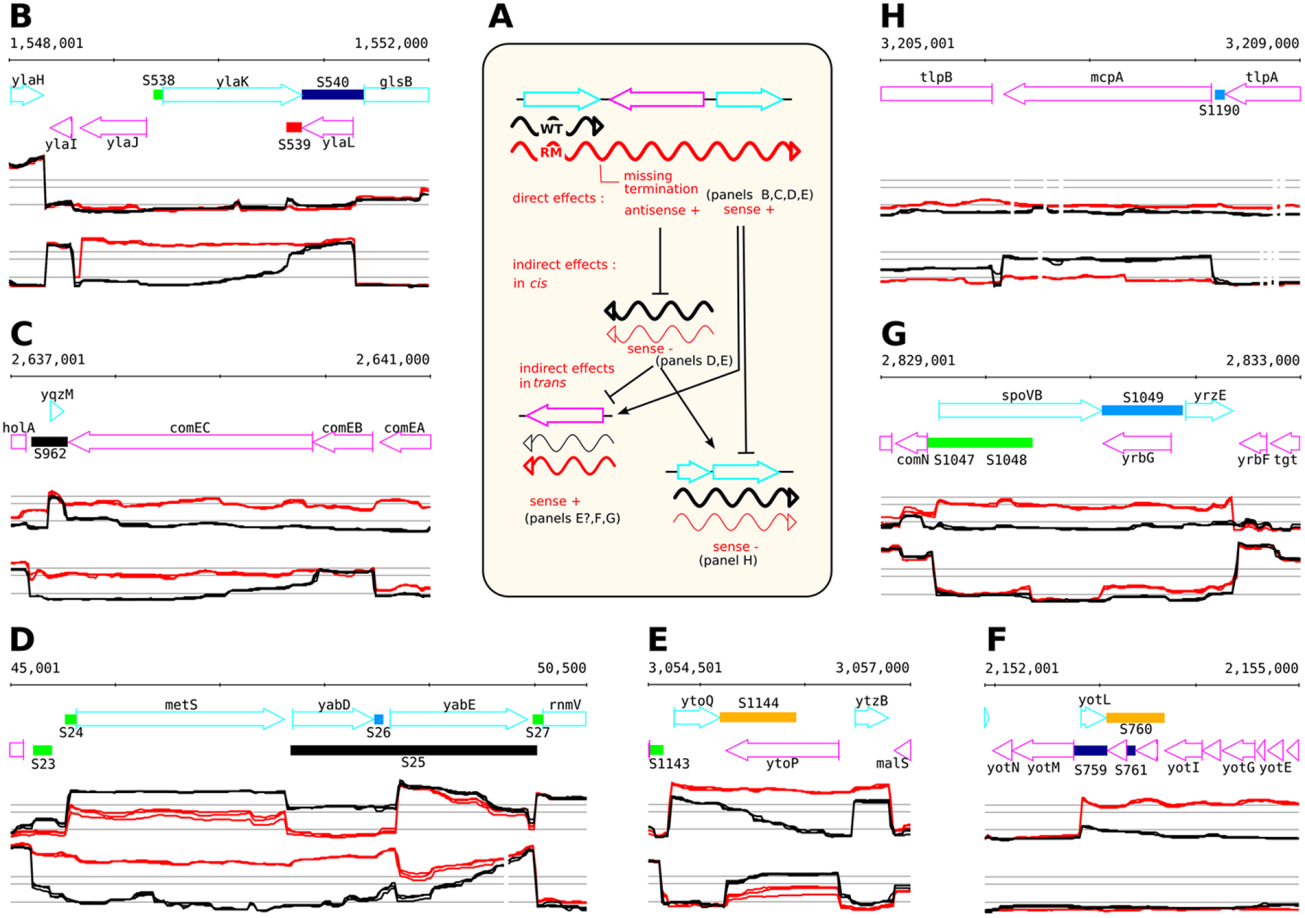
A typology of the different effects of *rho* deletion on the transcriptome. (A) Schematic illustration of the possible direct and indirect effects of Rho-controlled transcription. In WT, transcription terminates at Rho-controlled terminator (transcript is shown in black). In the RM, transcription is a result of missing termination (Rho-controlled transcript is shown in red). The transcript can be sense or antisense with respect to the orientation of the downstream genes. (B and C) Examples of direct up-regulation downstream of sites were termination is impaired in RM. (D and E) Examples of sense down-regulation facing up-regulated antisense transcripts arising from impaired termination in the RM. These down-regulations are presumably caused by indirect effects in *cis*. (F, G and H) Examples of up- or down-regulation occurring in chromosomal regions without visible impaired termination events. These are presumably caused by indirect effects in *trans*. Sections show annotated genome (top) and expression profiles on the (+) and (–) strands (mid and bottom sections, respectively). WT (black) and RM (red) profiles are shown. Expression profiles are from the *B*. *subtilis* expression data browser (http://genome.jouy.inra.fr/cgi-bin/seb/index.py).

Expression changes on the sense strand can be divided into three main categories (Fig 2A): direct up-regulation downstream of Rho-dependent termination sites; indirect *cis* effects by which an up-regulated antisense transcription affects expression of the overlapping gene on the opposite strand; and indirect *trans* effects resulting from regulatory cascades initiated by direct effects. Disentangling these three types of effects can be difficult.

Based on *B*. *subtilis* 1012 tiling arrays, up-regulation was detected for the sense expression levels of 456 (7.8%) native TRs, including 181 S-segments and 275 protein coding genes. Most of these events were due to altered termination of transcription (direct *cis* effect) as previously reported [17] (Fig 2B and 2C). In total, 90 up-regulated S-segments and 197 up-regulated protein coding genes clearly result from imperfect termination (termination read-through and/or lack of termination; Fig 2D and 2E). However, 26.3% (120/456) of the detected up-regulated native TRs were associated with at least two-fold increased expression levels immediately after the corresponding promoters (Fig 2F and 2G) and thus likely result from indirect *trans* effects. Similarly, down-regulation of 163 of 223 (73.1%) native TRs was associated with significantly decreased expression levels (RM/WT log2 ≤ -1) immediately after promoters (Fig 2H), which could reflect either decreased RNA synthesis, due to indirect *trans* effects, or increased RNA degradation.

Although the tiling array analysis indicated that genes targeted in RM cells by anti-sense transcripts were down-regulated more often than would be expected at random, this result could not be entirely confirmed with the RNA-Seq data. Thus, it is still unclear whether the up-regulated antisense strands in *B*. *subtilis* cells contribute globally to down-regulation of the sense strands, in addition to indirect *trans* effects.

### Rho inactivation affects the expression of genes from different functional categories

We obtained further insights into the potential physiological consequences of the altered expression profiles in the RM strains by analyzing the distribution of up- and down-regulated native TRs in terms of regulons and functional categories (sigma factor regulons as established in [17], other regulons, and functional categories, as designated in the *Subti*Wiki database [65]). The complete list of statistically significant associations based on the analyses of *B*. *subtilis* 1012 tiling array and NCIB 3610 RNA-seq data (Fisher exact test p-value ≤ 1e-4) is presented in S2 Table.

Over-representation of the SigD regulon (p-value 1e-59) among the down-regulated native TRs accounted for the strongest associations with known functional categories and regulons in both 1012 RM and NCIB 3610 RM strains. Most of the genes from functional category “motility and chemotaxis” were down-regulated in both RM strains, consistent with known functions of SigD. All genes from the Spo0A and CodY regulons, which were over-represented (p-value 6.59e-15 and 1.57e-17, respectively) among the down-regulated TRs in both RM strains also belong to the SigD regulon. PBSX prophage genes and genes controlled by the specific sigma factor Xpf were down-regulated in the 1012 RM but not the NCIB 3610 RM strain.

The up-regulated protein-coding genes and S-segments exhibited weaker biases towards specific regulons and functional categories. Nonetheless, we observed over-representation of genes from the SigK and SigG regulons (p-value 4e-9 and 9.7e-5, respectively), active during sporulation, and genes from the close functional category of “sporulation proteins” (p-value 1.4e-7) [65] (S2 Table). These genes were not expressed in WT cells during exponential growth in rich LB medium. SigA genes were statistically under-represented (p-value 2e-9), but still accounted for a majority of this set of up-regulated genes. Over-representation of the SigB regulon (p-value 3.8e-7) was detected in the NCIB 3610 RM but not 1012 RM strain.

We performed a comparative proteome analysis of the BSB1 WT and RM strains (Materials and methods) to complement the transcriptome data. Membrane and cytosolic fractions were prepared separately to maximize the chances of protein identification. Protein Abundance Index (PAI) values, calculated from mass spectrometry data, were used to compare the two proteomes [66]. In total, 1,619 proteins were identified (see S1 Table for the PAI of identified proteins and S3 Table for raw proteome data), corresponding to 38% of the protein-coding genes. The log2 PAIs of detected proteins correlated with the abundance of cognate mRNAs both in WT and RM strains (Pearson correlation coefficients > 0.60). We evaluated the effect of Rho inactivation on the proteome using the same two-fold cut-off as for the transcriptome: abundance increased for 157 proteins and decreased for 101 proteins in RM, with 85 proteins detected only in RM and 38 detected only in WT cells (S1 and S3 Tables). The proteome analysis confirmed the strong down-regulation of the SigD regulon in the absence of Rho: 42 of 44 SigD-controlled proteins detected in the WT proteome were under-represented in the RM proteome (Table 1). SigD protein itself was not detected in any proteome. The observed decrease of protein abundance in RM cells was variable and reached 26-fold in the case of the HemAT protein.

**Table 1:** SigD-dependent proteins identified by LC-MS/MS analysis in *B*. *subtilis* WT and RM cells.

Protein	PAI ^a^		PAI ratio RM/WT	mRNA ratio^b^ RM/WT	Function ^c^
	WT	RM			
FliH	0.23	nd	-	0.43	flagellar assembly protein
LytF	0.08	nd	-	0.12	major autolysin
TlpC	0.26	nd	-	0.37*	methyl-accepting chemotaxis protein
HemAT	1.57	0.06	0.04	0.21	heme-based aerotactic transducer
CheV	0.61	0.06	0.10	0.43*	CheA modulator
MotA	2.44	0.38	0.16	0.14	flagellar motor rotation
McpC	0.52	0.1	0.19	0.41	methyl-accepting chemotaxis protein
YfmT	2.03	0.5	0.25	0.15	unknown function/ vanillin dehydrogenase
MotB	6	1.5	0.25	0.14	flagellar motor rotation
TlpB	1.03	0.27	0.26	0.36	methyl-accepting chemotaxis protein
FliI	0.17	0.05	0.29	0.39	flagellar-specific ATPase
McpA	1.93	0.63	0.33	0.17	methyl-accepting chemotaxis protein
Hag	47.25	16.05	0.34	0.08	flagellin protein
YfmS	0.96	0.33	0.34	0.16	soluble chemotaxis receptor
FlgL	0.23	0.08	0.35	0.94	flagellar hook-filament junction protein
McpB	1.59	0.57	0.36	0.42	methyl-accepting chemotaxis protein
FliM	0.64	0.23	0.36	0.38	flagellar motor switch protein
LytD	0.13	0.05	0.38	0.30	major autolysin
TlpA	0.43	0.17	0.40	0.44	methyl-accepting chemotaxis protein
LytA	0.63	0.25	0.40	0.40	secretion of major autolysin LytC
FliY	0.91	0.37	0.41	0.34	flagellar C ring protein
FlgK	0.71	0.33	0.46	1.00	flagellar hook-filament junction protein
FlhA	0.92	0.44	0.48	0.31	part of the flagellar type III export apparatus
YjcP	0.57	0.28	0.49	0.52	unknown
FlgG	2.5	1.25	0.50	0.37	flagellar hook protein
SwrB	0.4	0.2	0.50	0.55	control of SigD activity/ flagellar type III secretion activator
FliF	1.18	0.62	0.53	0.55	flagellar basal-body M-ring protein
CheW	0.83	0.44	0.53	0.40	CheA modulator
YxkC	13.42	7.17	0.53	0.41	unknown
DltE	0.45	0.25	0.56	0.10	biosynthesis of teichoic acid
DltB	2.4	1.35	0.56	0.95	biosynthesis of teichoic acid
CheA	1.75	1.05	0.60	0.38	chemotactic signal regulator; two-component sensor kinase
DltA	3.81	2.38	0.62	0.84	biosynthesis of teichoic acid
PgdS	0.08	0.05	0.63	0.23	polyglutamic acid degradation
LytC	0.67	0.42	0.63	0.52	major autolysin
CheB	1.83	1.17	0.64	0.36	modification of methyl-accepting chemotaxis protein/ methylesterase
FliG	0.3	0.2	0.67	0.40	flagellar C ring protein
FliD	1.02	0.69	0.68	0.23*	polymerization of flagellin/ extracytoplasmic chaperone
DltD	3.02	2.11	0.70	0.88	biosynthesis of teichoic acid
CheR	0.77	0.6	0.78	0.69	modification of methyl-accepting chemotaxis proteins/ methyltransferase
FliS	0.5	0.4	0.80	0.26*	chaperone for flagellin export
FlhF	0.21	0.18	0.86	0.34	placement and assembly of polar flagella; signal recognition particle GTPase
LytB	0.31	0.31	1.00	0.47	autolysis; modifier protein of major autolysin LytC
LytE	0.2	0.24	1.20	0.98	major autolysin
DltC	4	5.5	1.38	0.91	biosynthesis of teichoic acid

^(a)^The PAI is the ratio between the total number of spectra obtained during the protein identification process on the theoretical number of peptides ranging between 800 to 2,500 daltons (Da) and detectable by mass spectrometry for a given protein [66] (S1 and S3 Tables); (nd) not detected.

^(b)^mRNA levels were computed on the normalized-aggregated data for the sense strand (Materials and methods, S1 Table). The presence of specific asRNA (S4 Table) is indicated by asterisk.

^(c)^Known function according to *Subti*Wiki [65].

In summary, transcriptome and proteome analyses documented notable alterations of the genome expression landscape in RM cells during exponential growth in rich medium. They highlight unscheduled expression of a number of sporulation genes and down-regulation of the SigD regulon, which could reflect important physiological changes. This prompted us to more thoroughly examine the impact of Rho inactivation on cell behavior during corresponding developmental programs.

### Rho inactivation impairs motility

Analysis of the transcriptome and proteome data showed that SigD-controlled genes were significantly down-regulated in RM derivatives of 1012 and NCIB 3610 strains (Table 1 and S1 Table). Further examination showed that genes belonging to the SigD regulon, but primarily expressed from SigA promoters [17, 65], displayed either a weak (for example, log2 RM/WT ≤ −1.0 for the *yjcP-yjcQ* operon) or intermediate (for example, log2 RM/WT ≤ −1.5 for the *fla-che* operon) decrease in expression. In contrast, genes exclusively controlled by SigD were strongly down-regulated (for example, log2 RM/WT = −2.8 in 1012; log2 RM/WT = −2.1 in NCIB 3610 for the *motA* gene). The expression level of *sigD* itself was lower in both RM strains (log2 RM/WT = −0.91 in 1012; log2 RM/WT = −1.69 in NCIB 3610) (S1 Table).

The down-regulation of 11 SigD-controlled genes was associated with the presence of asRNAs expressed above the cut-off level (S4 Table). Four of these asRNAs have been previously detected and annotated in BSB1 WT: S1367, S1403, S125, and S829 [17]. Expression of the non-annotated asRNAs facing the *yvyC-fliD-fliS-fliT-yvzG* operon and *cheV* and *flhO* genes was specific to RM (can be visualized on http://genome.jouy.inra.fr/cgi-bin/seb/index.py).

**Fig 3:**
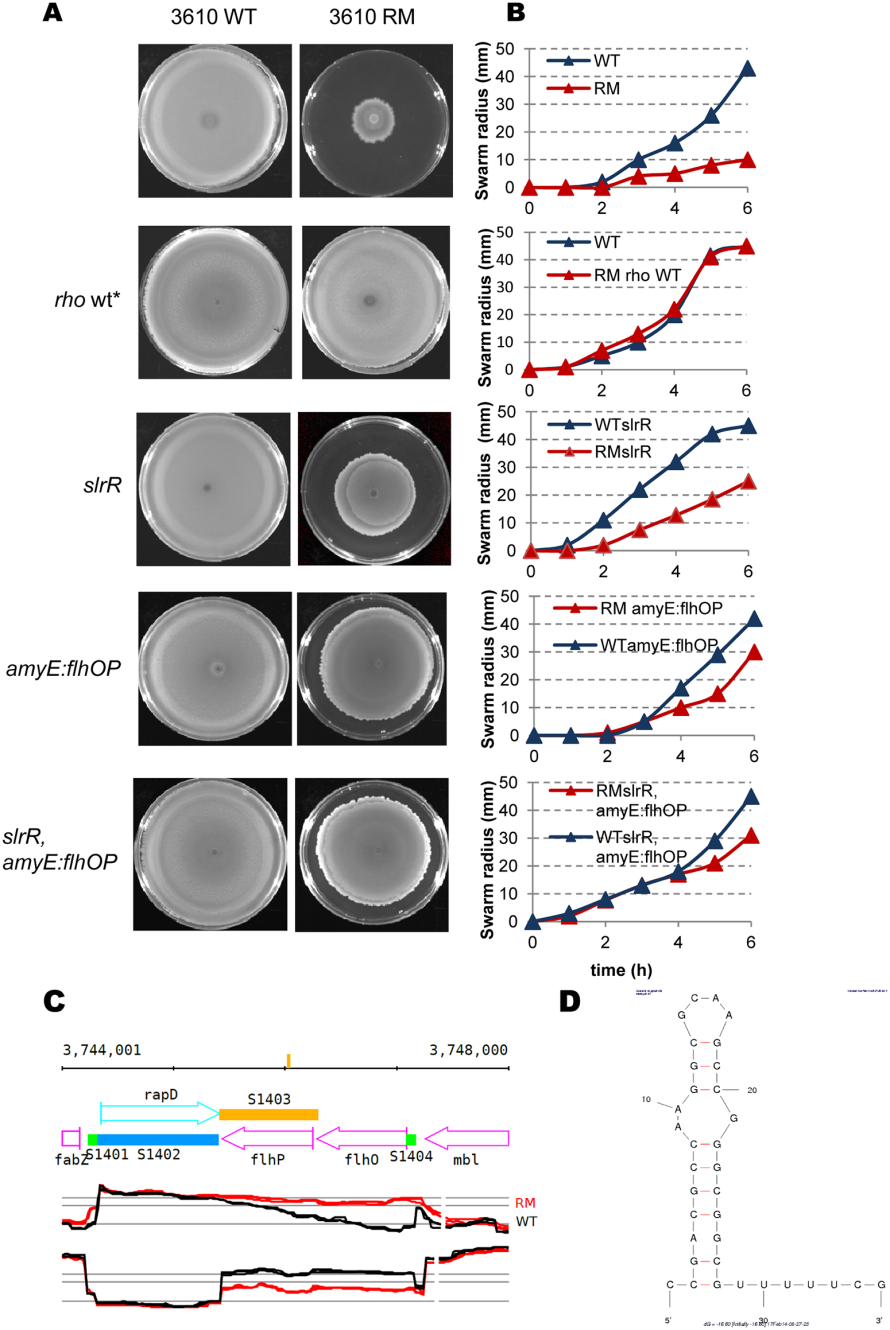
Impact of Rho inactivation on swarming motility of *B*. *subtilis* cells. (A) Motility defect of the NCIB 3610 RM cells can be partially suppressed by the deletion of *slrR* and ectopic expression of *flhO-flhP* genes. Bacterial cultures were grown to an OD_600_ 0.5, concentrated and spotted on the plate as described (Materials and methods). The images were acquired after 20 hours of incubation at 37°C. Each icon represents top-grown image of centrally inoculated Petri plate (diameter 9 cm) containing LB and 0.7% of agar. Relevant genotypes are indicated on the side of each image. The repaired back to the wild type NCIB 3610 RM is denoted as *rho* wt*. The experiment was reproduced at least five times and included three biological replicas for each strain. The results from the representative experiment are presented. (B) Quantitative swarming assay of the indicated NCIB 3610 (blue lines) and isogenic NCIB 3610 RM (red lines) derivative strains. Values represent the mean of at least five experiments. (C) Impact of *rho* deletion on sense and antisense transcription of the *flhO-flhP* operon in the *B*. *subtilis* 1012 cells. Expression profiles are from the *B*. *subtilis* expression data browser (http://genome.jouy.inra.fr/cgi-bin/seb/index.py). Vertical bar on the top line indicates position of predicted putative terminator (shown in D). Sections show annotated genome (top) and expression profiles on the (+) and (–) strands (mid and bottom sections). Wild type (black) and RM (red) profiles are shown. (D) MFOLD [83] predicted secondary structure (ΔG = −16, 30) within *flhP* asRNA.

Several phenotypes are known to be associated with expression of the SigD regulon of *B*. *subtilis* cells, in particular, motility, the capacity to synthesize flagella and to swim in liquid, or to swarm over a solid surface [67]. SigD is a key determinant of the phenotypic switch between motile and sessile states within the exponentially growing cell population [40]. Cells with a high level of SigD (SigD-ON) are motile and those with a low level of SigD (SigD-OFF) are sessile. We compared the undomesticated strain NCIB 3610 and its isogenic *rho* mutant to investigate the impact of Rho inactivation on the SigD-controlled motility phenotype, as laboratory strains of *B*. *subtilis* do not exhibit swarming motility [68]. Swarming of the NCIB 3610 RM strain was significantly impeded (Fig 3). We restored the wild type *rho* allele at the chromosome of the RM strain to prove that the observed deficiency was directly linked to Rho inactivation. Conversion of the NCIB 3610 RM derivative back to wild type (NCIB 3610 *rho* wt*) restored motility, showing that the motility-deficient phenotype of the NCIB 3610 RM strain was due to the deletion of *rho* (Fig 3A and 3B).

Complex mechanisms, including several regulatory feedback loops, control expression of the genes from the SigD regulon and *sigD* gene itself, thus determining the SigD-ON or SigD-OFF state [40]. In particular, the anti-sigma factor FlgM antagonizes SigD activity by direct binding to SigD and inhibition of its interaction with RNA polymerase [69]. Additionally, genes from the SigD regulon are negatively controlled by the SinR, SlrR, and SlrA transcription factors, acting as SinR-SlrR and SlrR-SlrA heterodimers or a SlrA/SinR/SlrR functional complex [70, 71]; global transcription regulator CodY [72]; stringent response regulator RelA [73]; and adaptive response regulator YmdB [74, 75]. Global regulator DegU acts as a repressor or activator of the SigD regulon, depending on its phosphorylation state [76]. Finally, SwrA and SwrB proteins positively control SigD [68, 77].

Expression of most of these regulatory genes was not significantly different between RM and WT cells, with the exception of lower expression of *swrB* (log2 RM/WT = −0.88 in 1012; log2 RM/WT = −1.61 in NCIB 3610) and higher expression of *slrR* (log2 RM/WT = 1.44 in 1012; log2 RM/WT = 3.16 in NCIB 3610) and *slrA* (under the two-fold cut-off level, log2 RM/WT = 0.967 in 1012; log2 RM/WT = 0.60 in NCIB 3610) genes. Increased expression of both *slr* genes was due to 3’ extensions: of the asRNA targeting the *epsA-O* operon for the *slrR* gene (see next section); and of the S1475 segment for the *slrA* gene (S2 Fig). Extension of the S1475 segment protects the *slrA* mRNA from enzymatic degradation [78]. An increase in *slrA* copy number down-regulates the *fla/che* operon containing the *sigD* gene and, consequently, the entire SigD regulon; this inhibition depends on active SlrR and SinR proteins [71, 78]. Thus, we tested whether the non-motile phenotype of RM cells was due to increased expression of *slrR* and/or *slrA*. Inactivation of the *slrR* gene, which disables both regulators [71], partially restored the motility of NCIB 3610 RM cells (Fig 3).

The observed homogenous down-regulation of genes exclusively transcribed from SigD promoters suggests direct inhibition of SigD activity. This prompted us to consider the possible implication of FlgM in the observed phenotype of the RM strain. The anti-SigD activity of FlgM is dose-dependent and transcriptionally and post-translationally controlled [79–81]. Transcriptome analysis did not reveal any changes of *flgM* expression in RM strains (S1 Table; http://genome.jouy.inra.fr/cgi-bin/seb/index.py). Post-translational regulation of FlgM is exerted via FlgM secretion from the cytoplasm by the flagellar export apparatus after assembly of the intermediate hook-basal body of flagellum [81]. Thus, SigD activity tightly correlates with the efficiency of FlgM secretion, which in turn depends on completion of the flagellar hook [67, 81, 82]. It is thus remarkable that expression of the *flhO*-*flhP* genes encoding the components required for hook completion was significantly lower in the RM strains (for the *flhO* gene, log2 RM/WT = −2.06 in 1012; log2 RM/WT = –1.62 in NCIB 3610). The decrease of *flhO-flhP* transcription correlates with the ~ 860 nucleotides (nt) long 3’-extension of the annotated S1403 asRNA (log2 RM/WT = 2.77 for the *flhO* asRNA in 1012, log2 RM/WT = 3.92 in NCIB 3610), (Fig 3C and S1 Fig). In the absence of Rho, transcription of S1403 extended through the RNA hairpin structure (ΔG = −16.6) [83] within the *flhP* gene (Fig 3C and 3D). The *flhP* asRNA, with the 3’-end matching the position of this hairpin, was detected by genome-wide 3’ end-mapping in the Rho-proficient *B*. *subtilis* PLBS802 strain [84]. In RM cells, the extended S1403 asRNA spreaded over the whole *flhO* gene, overlaped with the *flhO-flhP* promoter, and may have down-regulated expression of the *flhO-flhP* operon. This could impede the synthesis of flagellar hook, leading to FlgM accumulation in the cytoplasm and consequently, reduced expression of SigD-dependent genes, as observed for *B*. *subtilis flhO* and *flhP* mutants [82].

First, we tested this possibility by examining the contribution of FlgM to the motility-defective phenotype of the RM strain by inactivating the *flgM* gene. Deletion of *flgM* improved the motility of NCIB 3610 RM cells (S3 Fig). We next compensated the down-regulation of the *flhO-flhP* operon in RM cells by inserting a copy of the *flhO-flhP* operon expressed from its own promoter, into the *amyE* chromosomal locus of NCIB 3610 RM (NCIB 3610 RM *amyE*::*P*_*flhO*_*-flhO-flhP*). The expression of the *flhO-flhP* genes from the ectopic position improved the swarming motility of NCIB 3610 RM (Fig 3A and 3B). Subsequent inactivation of the *slrR* gene (NCIB 3610 RM *slrR*, *amyE*::*P*_*flhO*_*-flhO-flhP)* had an additive effect, but yet did not restore cell motility to the WT level (Fig 3A and 3B). This pinpoints the existence of additional factors that inhibit motility in RM cells.

Rho-controlled sense transcripts associated with *slrR* and *slrA* and the antisense transcript associated with *flhO-flhP* genes represent newly identified components of the regulatory network that control cell motility. These findings provide additional evidence that read-through of Rho-dependent terminators affecting the expression of downstream genes can propagate into regulatory networks and cause phenotypic changes.

### Rho inactivation impairs biofilm formation

The switch from the motile to sessile state in growing *B*. *subtilis* populations is associated with activation of an alternative developmental program, known as biofilm formation. Biofilms are multicellular aggregates assembled within a self-produced extracellular matrix. The main components of the biofilm matrix, exopolysaccharides (EPS) and amyloid-like protein fibers, are encoded by the 15-gene-long *epsA-O* operon and the *tapA-sipW-tasA* operon, respectively [85–87]. The global transcription regulator, Spo0A, indirectly controls the expression of matrix operons through the AbrB and SinI/SinR pathways [56, 88–92]. Expression of both operons is activated when Spo0A~P is present at low and/or intermediate levels and suppressed by high levels of Spo0A~P [47, 56, 88].

Motility genes are involved in the initial stages of air-liquid interface biofilm (pellicle) formation, but not in the development of architecturally complex colonies (colony biofilm) on an agar surface [93, 94]. Non-motile cells can also proceed to biofilm formation directly [39, 41, 61, 88, 95]. Thus, inactivation of Rho may affect the program of biofilm development due to altered SigD activity. In addition, up-regulation of *slrR* and *slrA* genes (S1 Table) could contribute to de-repression of the matrix operons and favor biofilm development in RM cells.

**Fig 4:**
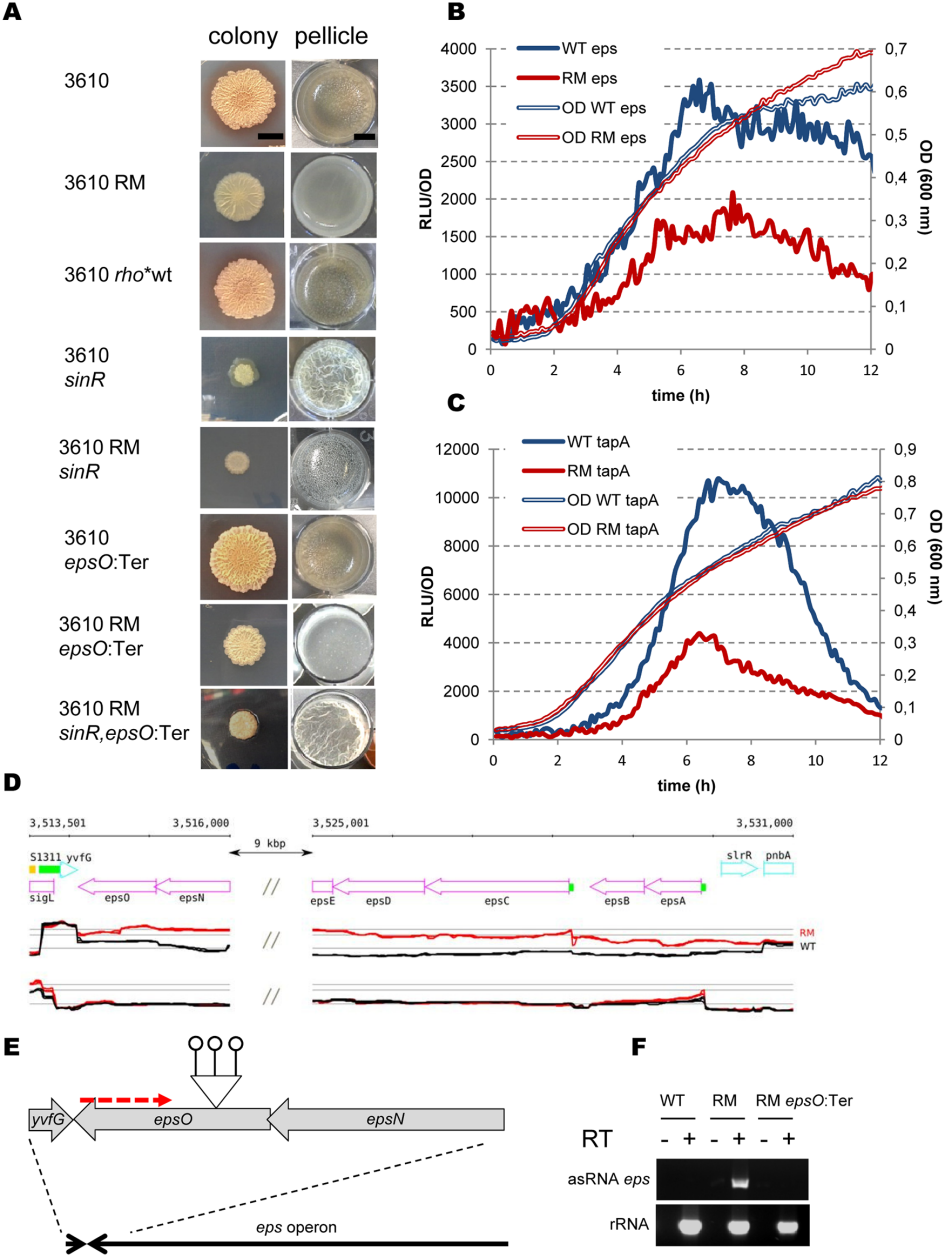
Rho inactivation negatively affects biofilm formation in *B*. *subtilis*. (A) Colony (left column) and pellicle (right column) biofilm formation by *B*. *subtilis* NCIB 3610 WT and RM strains. Relevant genotypes are indicated on the side of each image. The colony column shows individual colonies grown on MSgg agar medium for 72h at 30°C. The scale bar is 5mm. The pellicle column shows microtitre wells (diameter 1.5 cm) in which cells were grown in MSgg medium without agitation for 72h at 30°C. The scale bar is 5 mm. For the colony assay, 2μl of culture was spotted onto MSgg agar plate. For pellicle assay, 2μl of culture was added to 2ml of MSgg medium in a well of 24-well sterile microtiter plate. The experiment was reproduced at least five times including four replicas for each strain. Presented are the results from the representative experiment. (B and C) Rho inactivation decreases expression of biofilm-specific genes. Promoters of the operons *epsA-O* (B) and *tapA-sipW-tasA* (C) encoding exopolysaccharides and amyloid-like protein fibers, respectively, were fused to the butterfly luciferase gene *luc* and tested for transcription activity in *B*. *subtilis* BSB1 WT (blue lines) and RM (red lines) cells grown in liquid biofilm-stimulating medium MSgg as described in Materials and Methods. The thin double lines depict growth curves measured by optical density OD 600nm and the thick lines show relative luminescence readings corrected for OD. The unmarked individual points of the curves are the means of the values measured at the 5 min intervals in four independent isogenic cultures during the same experiment. The experiment was reproduced three times and the results of the representative one are shown. (D) *B*. *subtilis* genomic region corresponding to the *epsA-O* operon shows asRNA of ~15740 nt long starting from the *epsO* gene and overlapping the entire operon in 1012 RM strain. The double slash marks represent internal part of *eps* operon (*epsE-epsF-epsH-epsI-epsJ-epsK-epsL-epsM-epsN*) that is not shown (the whole operon can be visualized on http://genome.jouy.inra.fr/cgi-bin/seb/index.py). Sections show annotated genome (top) and expression profiles of WT (black) and RM (red) on the (+) and (–) strands (mid and bottom sections). (E) Schematic illustration of the construction of NCIB 3610 *epsO*:Ter insertion mutant. Bars tipped with open circles and red dotted arrow denote Rho-independent terminators and *eps* asRNA respectively; elements not to scale. (F) RT-PCR analysis of antisense transcription of *eps* operon region (top section) in the NCIB 3610 WT and RM strains. RT-PCR analysis of rRNA (bottom section) was used as a control. Synthesis of cDNA was performed using 50 ng of total RNA as template (Materials and methods). Reactions were performed with (+) and without (-) reverse transcriptase (RT). PCR was done with oligonucleotides specific for *eps* asRNA within *epsK* gene (asRNA *eps*) and for rRNA (rRNA) (S7 Table).

We investigated the consequences of Rho inactivation on biofilm formation by comparing the dynamics of pellicle formation by the NCIB 3610 WT and RM strains in biofilm-promoting MSgg medium, as well as their capacity to develop architecturally complex colonies on an agar surface. The NCIB 3610 WT strain formed thick, robust pellicles and exhibited complex colony architecture as described in the literature [96] (Fig 4A). In contrast, the isogenic RM strain formed only thin pellicles and flat unstructured colonies, which did not attain a phenotype similar to that of the wild type biofilms (Fig 4A). There were no differences in the biofilm phenotypes between the NCIB 3610 WT and *rho*-restored NCIB 3610 *rho* wt* strains (Fig 4A). This shows that the biofilm-deficient phenotype of the NCIB 3610 RM strain is primarily due to the deletion of *rho*, similarly to the motility defect.

The defective architecture of colonies formed by the NCIB 3610 RM strain suggested that inefficient biofilm formation was not solely due to down-regulation of the SigD-regulon [43, 93]. We thus examined biofilm formation by the strain NCIB 3610 RM *amyE*::*P*_*flhO*_*-flhO-flhP*. Indeed, ectopic expression of the *flhO-flhP* operon, which improved motility of the NCIB 3610 RM strain, did not improve biofilm formation (S4 Fig).

We next compared expression of the matrix genes between the WT and RM strains, using transcriptional fusions of the *epsA* and *tapA* promoters with the firefly luciferase (*luc*) gene [97], to gain insight into the impaired capacity of RM cells to form biofilms. These fusions were introduced at the native *eps* or *tapA* chromosomal loci of BSB1 WT and RM strains. We monitored luciferase activity during growth in liquid MSgg medium with constant aeration ([88], Materials and methods). We observed maximal expression of the *eps-luc* and *tapA-luc* fusions in WT cultures at the end of the exponential growth phase, in accordance with previously published data [88]. At the same time, both the *eps* and *tapA* promoters were significantly less active in the RM strain (Fig 4B and 4C), indicating inefficient de-repression of the matrix operons negatively controlled by SinR [56, 91]. We therefore expected that inactivation of SinR would restore biofilm formation by the RM strain. We examined biofilms formed by the NCIB 3610 *sinR* mutant and observed the formation of vigorous pellicles, colonies with an elevated surface, and increased production of mucoid substances, as reported previously [51, 56, 91] (Fig 4A). In contrast, the NCIB 3610 RM *sinR* strain formed fragile and shattered pellicles, resembling those of the NCIB 3610 *eps* mutants [43, 86, 98], and colonies which were morphologically different from the Rho-proficient NCIB 3610 *sinR* mutant (Fig 4A). Inactivation of AbrB, the second repressor of matrix operons in *B*. *subtilis*, did not restore biofilm formation by the NCIB 3610 RM or NCIB 3610 RM *sinR* mutant strains (S5 Fig). Therefore, relieving matrix operons of SinR- and AbrB-mediated repression is not sufficient to restore normal biofilm formation by the RM strain.

The transcriptome analysis revealed an additional factor that could potentially interfere with biofilm formation by *B*. *subtilis* RM cells. It is represented by an asRNA of ~15,740 nt, which starts near the 3’-end of the *epsO* gene, probably due to read through at an intrinsic terminator of the oppositely oriented *yvfG* gene, and overlaps the entire *epsA-O* operon (for *epsC* asRNA, log2 RM/WT = 3.01 in 1012 and log2 RM/WT = 4.79 in NCIB 3610; Fig 4D, S1 Table).

We tested whether the activity of *eps* asRNA contributes to the impaired biofilm formation of RM cells by blocking its synthesis. This was achieved by insertion of three Rho-independent transcription terminators within the *epsO* gene, with the active orientation blocking the synthesis of *eps* asRNA (Materials and methods, Fig 4D and 4E). RT-PCR confirmed that the synthesis of *eps* asRNA was abolished in the NCIB 3610 RM *epsO*:Ter strain (Fig 4F). Previously, the *epsO* gene was shown to be dispensable for pellicle formation [99]. Indeed, the NCIB 3610 derivative carrying the *epsO*:Ter insertion did not display any defect in biofilm formation (Fig 4A). The NCIB 3610 RM *epsO*:Ter strain had somewhat stronger pellicles and more complex colony biofilms than the parental RM strain (Fig 4A). Simultaneous prevention of antisense transcription and de-repression of the matrix operons in the NCIB 3610 RM *sinR*, *epsO*:Ter strain greatly improved development of pellicles and colony biofilms, which were similar to those formed by the NCIB 3610 *sinR* mutant (Fig 4A). These results demonstrate that Rho-controlled *eps* asRNA negatively affects EPS production.

Altogether, our results show that inactivation of Rho results in impaired biofilm formation. This phenomenon is mainly due to inefficient de-repression of both matrix operons and the negative effect of the *eps*-specific asRNA on the expression of the *eps* genes.

### Rho inactivation perturbs Spo0A phosphorelay

Comparative transcriptome and proteome analysis of *B*. *subtilis* WT and RM cells revealed that Rho inactivation led to perturbations of the multi-component phosphorelay system responsible for Spo0A phosphorylation [48, 49], (Table 2; S6 Fig).

**Table 2:** The components of Spo0A phosphorelay differently expressed in *B*. *subtilis* WT and RM cells.

Gene	Sigma factor/ regulon	mRNA ratio^a^ RM/WT	Qvalue	Transcription profile modification^b^	PAI^c^		PAI ratio RM/WT	Function
					WT	RM		
***Kinases***								
*kinA*	SigH/Spo0A	1.22	1.00E-03	nm	nd	0.05	-	sensor kinase
*kinB*	SigA/CodY	4.06	3.55E-07	no termination	nd	0.21	-	sensor kinase
*kinC*	SigA	0.80	2.00E-03	nm	0.43	0.26	0.61	sensor kinase
*kinD*	SigA	0.92	6.30E-02	nm	0.19	0.13	0.68	sensor kinase
*kinE*	SigH	1.38	1.37E-04	nm	nd	0.15	-	sensor kinase
***Proteins controlling kinase activity***								
*sivB*	SigA/AbrB; LutR ; DegU	0.51	5.39E-07	low activation	nd	nd	-	Inhibitor of KinA
*sivA*	SigA/AbrB	0.43	1.17E-06	low activation	nd	nd	-	Inhibitor of KinA
*kbaA*	SigA/AbrB	4.10	4.47E-07	3’-extention of upstream RNA	nd	nd	-	positive KinB effector
***Phosphotransferases***								
*spo0B*	SigA	2.27	1.89E-06	3’-extention of upstream RNA	0.17	0.50	2.94	phospho- transferase
*spo0F*	SigH/Spo0A	1.86	4.24E-06	nm	0.22	0.33	1.50	phospho-transferase
***Ultimate target***								
*spo0A*	SigA;SigH/Spo0A	0.71	2.00E-03	nm	0.62	0.89	1.44	response regulator
***Phosphatases and their modulators***								
*yisI*	SigA	5.46	2.43E-06	high activation	nd	nd	-	Spo0A~P phosphatase
*rapA*	SigA/CodY; ComA;Spo0A	4.44	4.82E-06	no termination	nd	0.54	-	Spo0F~P phosphatase
*phrA*	SigA/CodY; ComA;Spo0A	5.73	1.20E-06	no termination	nd	nd	-	RapA inhibitor
*rapB*	SigA;SigD	0.60	1.45E-05	low activation	0.45	0.33	0.73	Spo0F~P phosphatase

^(a)^mRNA levels were computed on the normalized-aggregated data for the sense strand (S1 Table, Materials and methods)

^(b)^modifications of expression profiles can be visualized on http://genome.jouy.inra.fr/cgi-bin/seb/index.py; (nm) no visible modifications

^(c)^The PAI is the ratio between the total number of spectra obtained during the protein identification process on the theoretical number of peptides ranging between 800 to 2,500 Da and detectable by mass spectrometry for a given protein [66] (S3 Table);

(nd) not detected.

Among the genes coding for five sensor histidine protein kinases (KinA—KinE), which are at the basis of the Spo0A phosphorelay, the *kinB* gene was strongly upregulated in RM cells (log2 RM/WT = 2.02; Table 2). We also detected the KinB protein in the RM, but not WT proteome, consistent with the transcriptome data. Analysis of the transcription profiles of WT cells during exponential growth revealed that *kinB* mRNA level was not constant across the gene but showed a marked down-shift at approximately one-third part of the open reading frame. In contrast, we did not observe this down-shift in RM cells (http://genome.jouy.inra.fr/cgi-bin/seb/index.py). The level of gene expression is aggregated into a single value computed as the median for probes within the transcription region [17]. Thus, the presence of the down-shift strongly reduces the value of *kinB* mRNA expression in the WT relative to RM cells. The mechanism of an intragenic down-shift within *kinB* will be discussed later. RNA levels of the other kinase genes were not significantly affected in the RM strain (Table 2). However, the KinA and KinE proteins were detected in the RM but not the WT proteome, whereas the KinC and KinD kinases were detected at slightly decreased levels in the RM proteome.

The transfer of the phosphoryl group from sensor kinases to Spo0A is catalyzed by two phosphotransferases, Spo0F and Spo0B [48]. Both *spo0F* and *spo0B* transcripts were up-regulated in RM (log2 RM/WT = 0.895 and 1.185, respectively). The amounts of Spo0F and Spo0B proteins were also increased in the RM proteome (1.5- and 2.9-fold, respectively), consistent with the transcriptome data.

In addition to the main phosphorelay components, the expression of several genes encoding accessory proteins was modified in the absence of Rho. Transcript level of the *kbaA* gene, which encodes a positive effector of KinB [100], was higher in RM cells (log2 RM/WT = 2.04) due to the 3’ extension of the upstream *salA* mRNA. Transcript levels of the *sivA* and *sivB* genes, encoding factors that negatively control the level of Spo0A~P through inhibition of KinA autophosphorylation [101], were lower in RM cells (log2 RM/WT = −1.22 and −0.97, respectively). For both genes, this was apparently due to lower activity of the corresponding promoters (indirect *trans* effect).

The genes of the *rapA-phrA* operon, encoding RapA phosphatase, which specifically dephosphorylates Spo0F~P, and its inhibitor, the PhrA peptide [102], were equally up-regulated in the RM strain (log2 RM/WT = 2.15 and 2.52, respectively). We also detected higher levels of RapA in the RM proteome, consistent with the transcriptome data. The up-regulation of *rapA-phrA* mRNA levels in RM cells was associated with the disappearance of the down-shift within the *rapA* transcript observed in WT cells, similar to *kinB* transcription. In contrast, RapB, the second phosphatase active on Spo0F~P, was down-regulated in the RM strain, apparently due to lower activity of the *rapB* promoter. Transcript levels of the *yisI* gene, encoding a phosphatase specific for Spo0A~P [103], increased in the RM strain (log2 RM/WT = 2.50). The remaining components of the *B*. *subtilis* phosphorelay system were not significantly affected by the lack of Rho (S1 and S3 Tables) and are not reported in Table 2.

**Fig 5:**
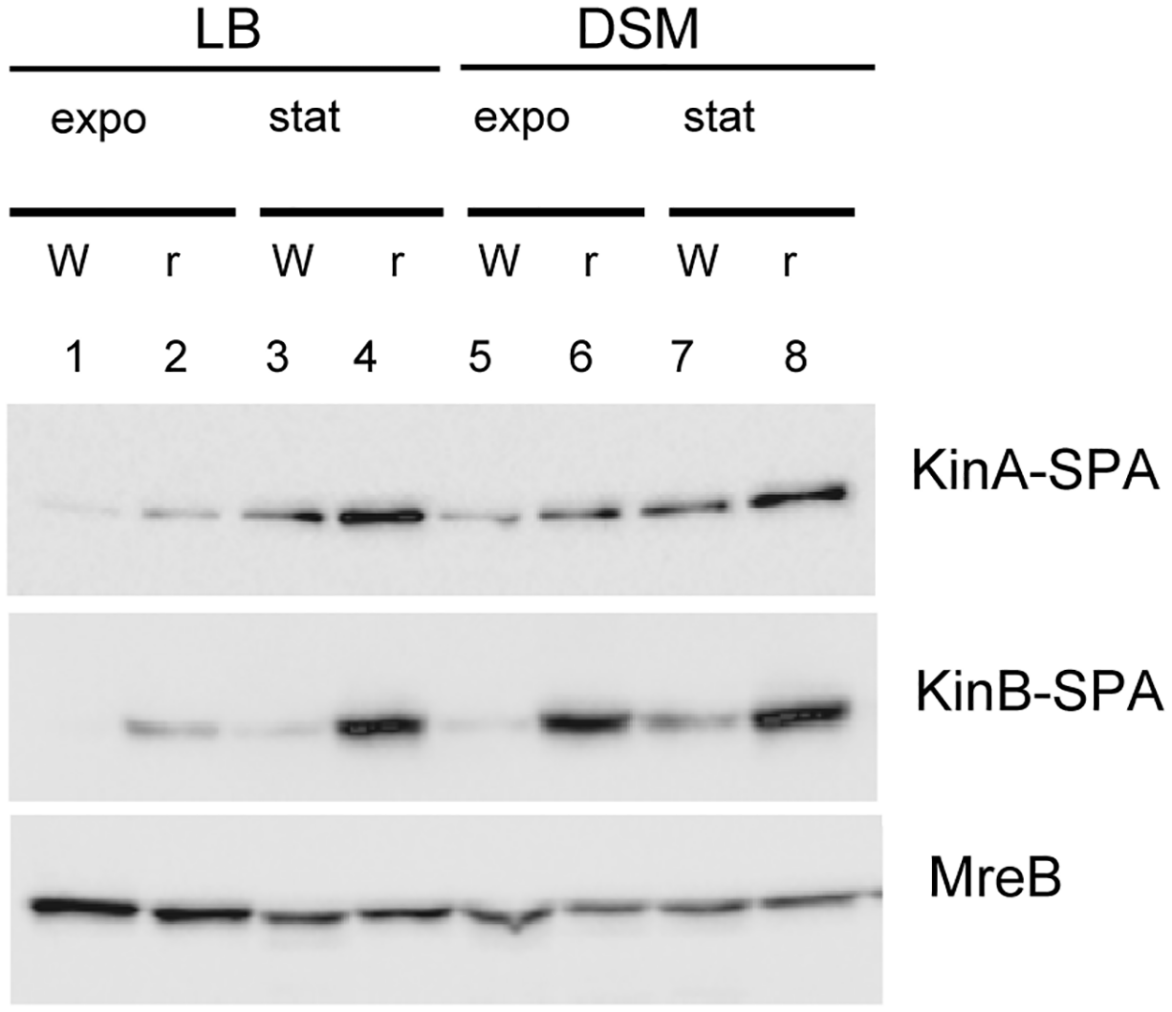
Rho inactivation increases expression of KinA and KinB kinases. WT (W) and RM (r) cells containing *kinA*-SPA or *kinB*-SPA translational fusions at natural chromosomal loci were grown in LB (lanes 1–4) or sporulation-inducing DS medium (lanes 5–10) to mid-exponential (expo; OD_600_ ∼ 0.5) or stationary (stat; OD_600_∼ 1.5) phases and analyzed for KinA and KinB proteins using ANTI-FLAG M2 monoclonal antibodies. Equal amounts of protein were loaded onto the gel as quantified by the Bradford assay. To control equilibrium between the samples, total protein extracts from cells with *kinB*-SPA fusion were analyzed for MreB protein using anti-MreB specific antibodies.

We translationally fused KinA and KinB proteins with the SPA peptide and compared the levels of SPA-tagged proteins in WT and RM cells to assess expression of these main sensor kinases at different growth stages. RM cells contained higher levels of KinA and KinB proteins than WT cells during the exponential and stationary phases of growth in LB (Fig 5). The effect was more prominent for KinB, as no or very little protein was detected in WT cells grown in LB. The propagation of cells in sporulation-inducing DS medium stimulated the synthesis of both kinases with a prevailing effect in RM cells, especially for KinB (Fig 5).

Taken together, these results highlight important changes in the expression of the multi-component phosphorelay system controlling the phosphorylation state of Spo0A in RM cells. The gradual activation of Spo0A~P by sequential phosphorylation might be shifted towards higher phosphorylation levels in the absence of Rho, given the known functions of the up- and down-regulated genes in this process.

### Rho inactivation increases Spo0A phosphorylation

We sought to experimentally establish whether the observed changes of phosphorelay in RM cells results in modification of the phosphorylation level of Spo0A~P as this could contribute to their defect in biofilm formation. Indeed, a high level of Spo0A~P induces suppression of the matrix operons [47, 56, 88]. During the transition to stationary phase, accumulating Spo0A~P increases *spo0A* gene expression via several positive feedback loops [104] and a transcription switch from the SigA-dependent vegetative promoter to the SigH-controlled sporulation-specific promoter [105]. We postulated that a high level of Spo0A~P in RM cells would lead to detectable changes of *spo0A* expression. The real-time kinetics of *spo0A* expression was previously analyzed at a population-wide level using the *luc* reporter gene fused, in-frame, to the *spo0A* start codon at its natural locus [97]. The construction monitors the activity of both *spo0A* promoters while maintaining an intact copy of the *spo0A* gene [97]. We used this *spo0A-luc* fusion to compare *spo0A* expression between the WT and RM strains.

**Fig 6:**
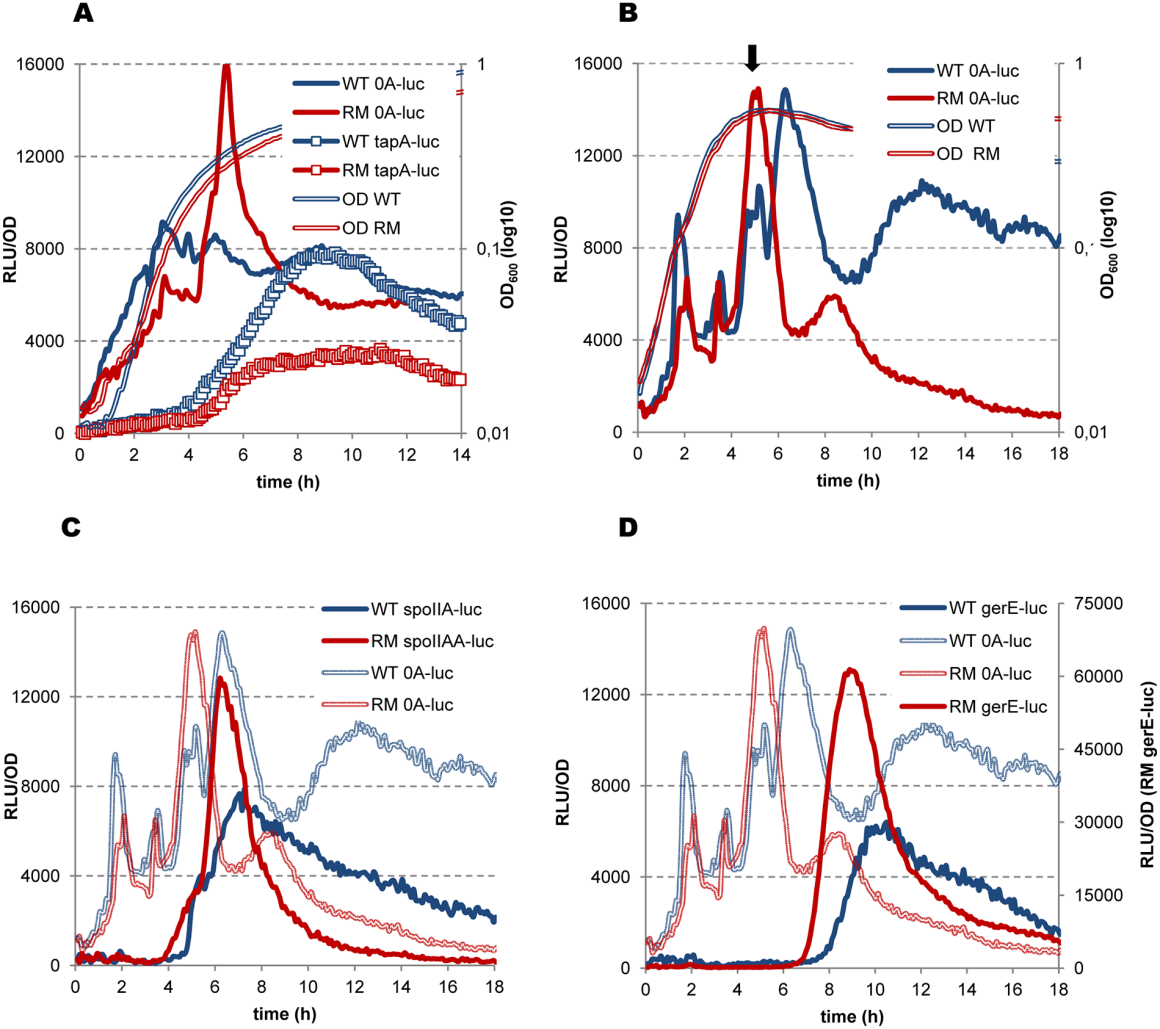
Rho inactivation increases Spo0A phosphorylation. *B*. *subtilis* BSB1 WT (blue lines) and RM (red lines) cells bearing transcription fusions of luciferase gene *luc* with the promoters of *spo0A* (A and B; solid lines), *tapA* (A; lines with squares), *spoIIAA* (C) and *gerE* (D) genes were analyzed for Luc expression during growth in biofilm-promoting MSgg medium (A) and sporulation-inducing DS medium (B, C and D) as described in Materials and Methods. Measurements were taken every 5 minutes after cells inoculation in media at optical density OD_600_ ∼0.025 (time point 0). For each strain, plotted are the mean values of luminescence readings corrected for OD from four independent cultures analyzed simultaneously. In (A and B), double-lined curves depict characteristic growth kinetics of cells measured by OD 600nm. In (B), arrow indicates entry in sporulation (T0) as established in [96]. In (C) and (D), shadowed double-lined curves reproduce kinetics of *spo0A* expression established in (B) during the same experiment. The experiment was reproduced at least three times. The results from the representative experiment are presented.

Initially, we analyzed the cells growing in biofilm-promoting MSgg medium, in which Spo0A~P accumulates to an intermediate level, stimulating de-repression of the matrix operons [58, 88]. We followed this event by simultaneous analysis of *tapA-luc* expression. The expression of *spo0A* in WT cells gradually increased during exponential growth and then remained relatively constant, producing a few weakly oscillating peaks (Fig 6A). At the end of exponential growth, one of the *spo0A* expression peaks coincided with activation of the *tapA-sipW-tasA* operon, indicating that the cells accumulated an appropriate Spo0A~P level. In RM cells, *spo0A* expression was lower during exponential growth than in WT cells, but exhibited a spike at the end of exponential growth (Fig 6A). Such a burst of *spo0A* activity might reflect activation of the sporulation-specific, SigH-dependent *spo0A* promoter by a high level of Spo0A~P [105]; the transition phase-specific SigH factor has been shown to be active in MSgg medium on other promoters [89]. Indeed, the spike of *spo0A* activity in RM cells correlated with decreased *tapA-sipW-tasA* expression, known to be suppressed by a high level of Spo0A~P [87, 106].

Next, we assessed *spo0A* expression in WT and RM cells grown in sporulation-inducing DS medium. In WT cells, *spo0A* expression was characterized by several pulses during the exponential and stationary growth phases, closely resembling previously reported *spo0A* expression kinetics (Fig 6B), [97]. A double-headed peak of *spo0A* expression observed at the moment of growth arrest has been previously shown to mark entry of the cells into sporulation (T0), as it coincides with activation of the early sporulation genes (Fig 6B), [97]. This peak would reflect a sporulation-inducing high threshold level of Spo0A~P [107]. Inactivation of Rho had no significant effect on *spo0A* promoter activity during exponential growth of RM cells but modified it at T0, when *spo0A* expression peaked at a higher level than in WT cells. This spike in the activity of the spo0A promoter at T0 was highly reproducible in RM cells and most likely resulted in a higher Spo0A~P level than in WT cells.

We further established an increase in the level of Spo0A~P in sporulating RM cells by following luciferase expression from the SigH-dependent promoter of the *spoIIAA-AB-sigF* operon, which is activated by a high level of Spo0A~P [57, 108]. In both WT and RM strains, *spoIIA-luc* induction coincided with pulses of *spo0A* activity, attributable to high Spo0A~P levels (Fig 6C). However, *spoIIA-luc* expression in the RM culture initiated about one hour earlier and was notably more efficient than in WT cells. This indicates that a sub-population of cells, in which Spo0A~P reached the required threshold to activate early sporulation genes, was higher in the RM culture.

We investigated whether the changes of *spo0A* activity in RM cells propagate further into the sporulation-specific cascade of gene expression by analyzing the expression of the *gerE* gene, which depends on the late mother cell-specific sigma factor, SigK, thus reflecting the final steps of sporulation [109]. The expression of *gerE-luc* in RM cells occurred within a narrow pulse starting ~1.5 hours earlier and reaching a ~10-fold higher maximum than in WT cells (Fig 6D; note different ordinates for the WT and RM *gerE-luc* expression curves). Such kinetics would account for more synchronous sporulation in the RM population, most probably due to efficient initiation of the process by high Spo0A~P.

In summary, different expression patterns of *spo0A* and Spo0A-regulated genes in WT and RM cells account for more efficient phosphorylation of Spo0A in the absence of Rho, both under biofilm- and sporulation-promoting growth conditions. In RM cells, the rapid increase of Spo0A~P to a high level in MSgg medium could inhibit matrix gene transcription and thus impair biofilm development, whereas in DS medium, higher Spo0A~P levels would trigger sporulation earlier and in a larger sub-population of cells.

### Rho inactivation increases the efficiency of *B*. *subtilis* sporulation

**Fig 7:**
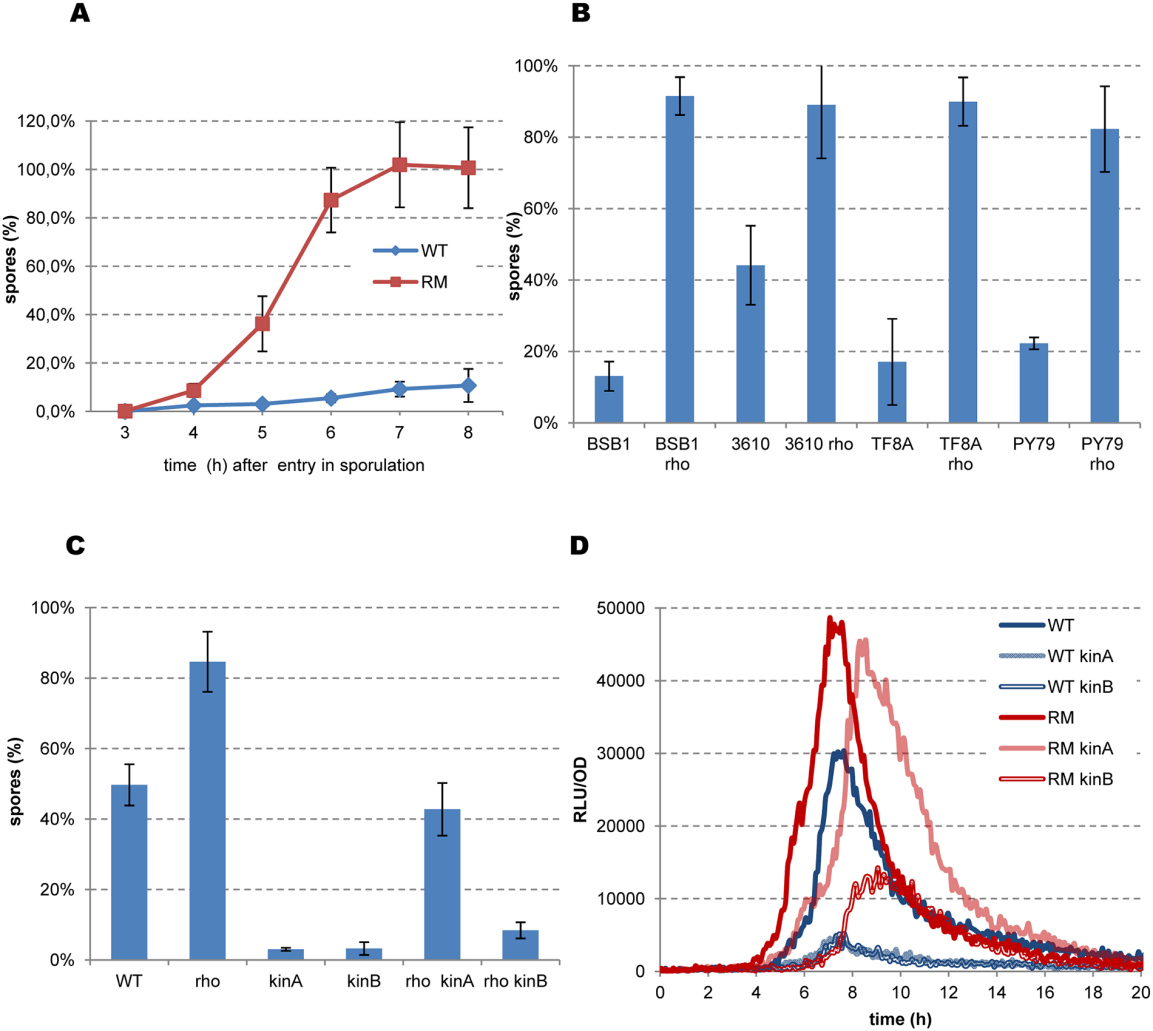
Rho inactivation accelerates sporulation of *B*. *subtilis* cells. (A) Sporulation kinetics of *B*. *subtilis* BSB1 WT and RM cells. Cells were grown in sporulation-inducing DS medium at 37°C with vigorous aeration up to OD_600_ 1.5. Starting from this time-point (sporulation point T0), samples were taken from cultures each hour and analyzed for spores by heating at 75°C as described in Materials and Methods. Sporulation efficiency was estimated as proportion of viable cells in the heated and unheated cultures. Plotted are the average values and standard deviations from four independent experiments each incorporating three biological replicas of each strain. (B) Sporulation efficiency of the BSB1, PY79, NCIB 3610 and TF8A WT strains and their respective RM derivatives at sporulation point T7. Cells were grown in DS medium during seven hours after T0 and analyzed for heat resistant spores as described in (A). (C) Sporulation efficiency of the BSB1 WT, BSB1 RM strains and their respective *kinA* and *kinB* mutants. Cells were inoculated in DS medium at OD_600_ 0.05, incubated at 37° during 20 hours and analyzed for spores as in (A). Totally, nine biological replicas of each strain were analyzed for (B) and twelve replicas for (C) in three independent experiments. Plotted are the average values with standard deviation error bars. (D) Kinetics of luciferase expression from *spoIIA-luc* fusion in the BSB1 WT (blue lines), BSB1 RM (red lines) and their respective *kinA* (light lines) and *kinB* (double lines) derivatives during growth in DS medium as described in Materials and Methods. For each strain, plotted are the mean values of relative luminescence readings corrected for OD from four independent cultures analyzed simultaneously. The experiment was reproduced at least three times. The results from the representative experiment are presented.

We then assessed whether the effects of the Rho mutation on the expression of sporulation genes leads to more productive sporulation. The laboratory *B*. *subtilis* 168-related strains are sporulation-proficient and could thus be used for this analysis. We used the exhaustion method to induce sporulation. The first heat-resistant spores were detected in BSB1 RM cultures four hours after entry into sporulation (T4), and by T7, almost 100% of the RM cells had formed spores (Fig 7A). Less than 20% of the WT cells had produced spores by the same timepoint, reflecting the well-known dichotomy of sporulation in *B*. *subtilis* [104, 109, 110]. We performed the same experiment using other *B*. *subtilis* strains: non-domesticated NCIB 3610; TF8A, a phage-cured derivative of 168; and PY79, a laboratory prototroph strain genetically distant from 168 [64, 111]. Deletion of the *rho* gene accelerated sporulation in all genetic backgrounds and most of the RM cells produced mature spores by T7(Fig 7B).

We examined whether *rho* deletion can suppress sporulation defects of the *kinA* and *kinB* mutants to formally link more efficient sporulation by RM cells to increased activity of the Spo0A phosphorelay. KinA is commonly considered as the main sporulation kinase, as its inactivation severely inhibits this process. The inhibitory effect of *kinB* mutations is more variable and apparently depends on the genetic background of the cells [53, 112, 113]. We cultured the *kinA* and *kinB* mutants of BSB1 and PY79 under sporulation conditions and reproduced both reported trends: the *kinA* mutation strongly reduced sporulation in both strains, whereas the inhibitory effect of the *kinB* mutation was strong in BSB1 and weak in PY79 (Fig 7C and S7 Fig). We used BSB1 derivatives for further experiments to remain consistent with the gene expression analysis, although the efficiency of sporulation was generally lower in the BSB1 than PY79 background.

Inactivation of Rho improved the sporulation of BSB1 *kinA* and *kinB* mutants, although to different degrees: an increase of ~15 fold in RM *kinA* and ~2.5-fold in RM *kinB* strains relative to the BSB1 *kinA* and *kinB* mutants (Fig 7C). Similarly, the *rh*o mutation preferentially rescued the sporulation defect of the PY79 *kinA* mutant, although the effect was relatively small (S7A Fig). Inactivation of Rho in the double *kinA kinB* mutants had no effect on the basal level of sporulation in either background (S7B Fig; data presented for the PY79 mutant derivatives). Altogether, the observed genetic interactions indicate that the stimulatory effect of *rho* deletion on sporulation mostly involves the KinB kinase, suggesting its increased role in the Spo0A phosphorelay system in RM cells.

We tested this hypothesis by analyzing the expression of the Spo0A~P-dependent *spoIIAA-AB-sigF* operon during sporulation of the kinase mutants. Expression of *spoIIA-luc* was similarly inhibited in BSB1 *kinA* and *kinB* mutants, indicating a strong decrease of Spo0A activity in the absence of either kinase (Fig 7D), corroborating the results of the sporulation assay in these strains. The expression of *spoIIA* in RM *kinA* cells was higher than the wild-type level, nearly reaching the maximum observed in RM cells (Fig 7D). However, *spoIIA* induction was delayed in RM *kinA* cells relative to RM cells (Fig 7D), which might underlie their different sporulation efficiencies (Fig 7C). Rho inactivation in the *kinB* mutant (RM *kinB*) also improved the expression of *spoIIA*, however it remained below the wild-type level (Fig 7D). The pattern of *spoIIA* expression in RM *kinB* cells thus correlates with their low sporulation efficiency. Altogether, these results indicate that the increased phosphorylation of Spo0A in the RM strain is mainly due to KinB.

### The *kinB* gene contains the Rho-dependent transcription terminator

**Fig 8:**
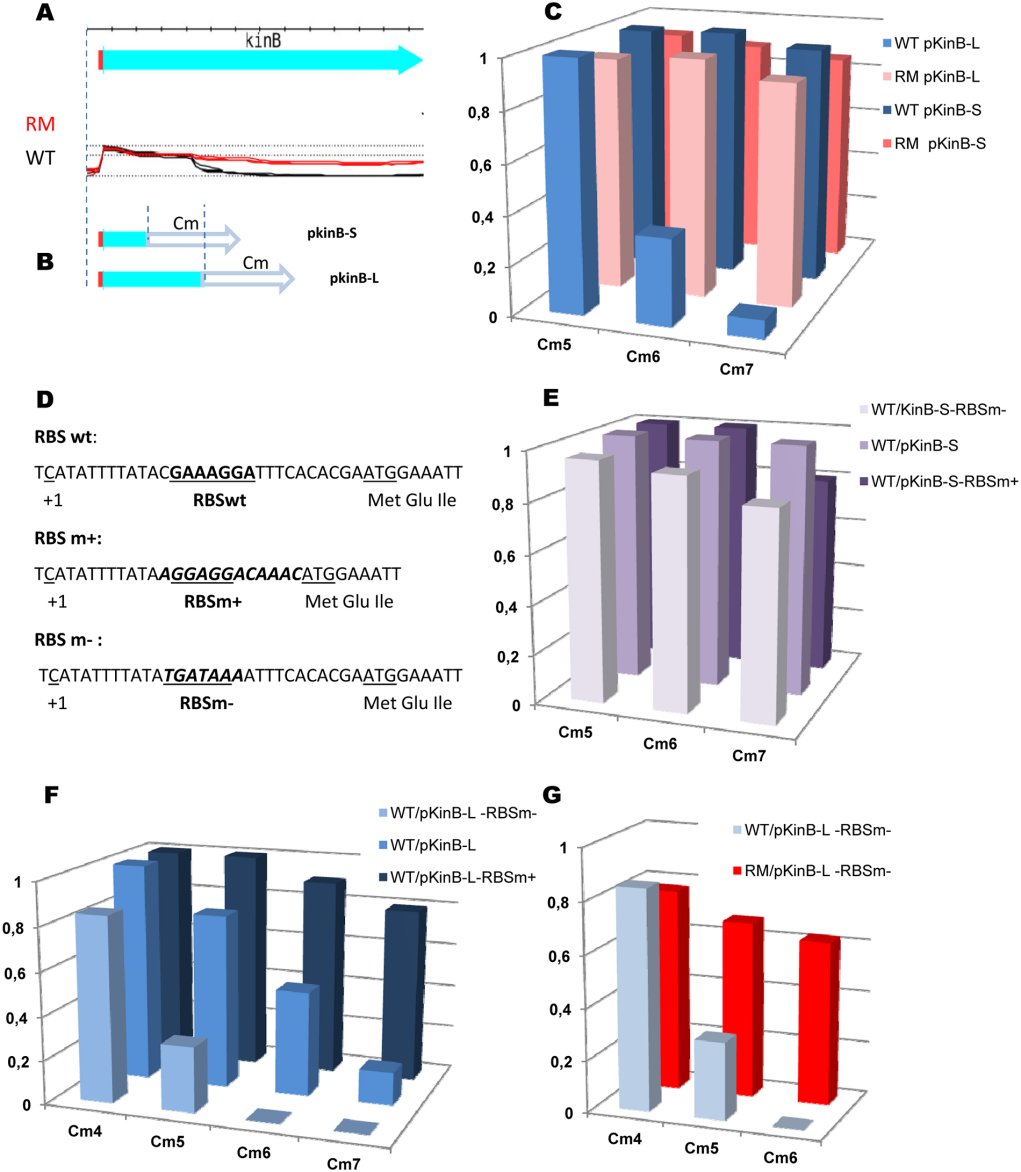
*B*. *subtilis kinB* gene contains intragenic Rho-dependent terminator. (A and B) Schema of the experimental design used for analysis of the *kinB* putative Rho-dependent terminator. (A) Cartoon of the *kinB* expression unit and the expression profiles of *kinB* in the WT (black) and RM (red) cells [17]. (B) Transcription initiation region (small red rectangle) and the 5’-terminal parts of *kinB* gene were cloned at the plasmid pGKV210 [116] upstream the promoter-less chloramphenicol-resistance gene (open arrow). The cloned fragments are delineated by the dotted lines. (C) Rho activity determines cellular resistance to chloramephenicol. *B*. *subtilis* BSB1 WT and RM cells containing pKinB-S*hort* (pKinB-S) and pKinB-L*ong* (pKinB-L) plasmids were grown to OD 0.5 and platted in sequential dilutions at the LB-plates containing or not chloramphenicol (Cm) at the indicated concentrations (μg/ml). Cm-resistant cells were scored after 24 hours of incubation at 37°C and compared to total number of viable cells. The bars represent average values from three independent experiments totally including twelve biological replicas for each strain. (D-G) Initiation rate of *kinB* translation negatively affects efficiency of Rho-dependent intragenic termination of *kinB* transcription. (D) Nucleotide sequence of the translation initiation regions (TIR) carrying native (RBSwt; [112]) and the modified strong (RBSm+) or weak (RBSm-) ribosome binding sites. The RBS sequences are bolded and underlined. The whole modifications of TIR sequences are bolded and in italics. The *kinB* transcriptional start (+1) and ATG codon are underlined. (E, F) *B*. *subtilis* BSB1 WT cells carrying pKinB-S or pKinB-L plasmids with different *kinB* RBS (RBSwt, RBSm+ and RBSm-) were analyzed for Cm-resistance as described in (C). (E) Modifications of *kinB* RBS have no effect on Cm-resistance when plasmids do not contain transcription terminator within *kinB* (pKinB-S). (F) In the presence of *kinB* transcription terminator, the level of Cm-resistance depends on the strength of *kinB* RBS (pKinB-L). (G) The pKinB-L-RBSm- plasmid with a weak RBS determines high level of Cm-resistance after Rho inactivation in RM cells. Each experiment depicted in (E-G) included three biological replicas of each strain and was repeated at least three times. The data for WT cells with pKinB-S and pKinB-L plasmids presented in (E) and (F) are independent from (C).

As mentioned above, transcriptome analysis revealed an abrupt down-shift within the *kinB* gene in WT, but not RM cells, explaining the higher expression of KinB in the absence of Rho (Fig 8A). The internal down-shift of *kinB* transcription is also observed in cells depleted of the RNaseY, RNase J1, or RNase III ribonucleases [114, 115], excluding its formation by the action of these enzymes. Analysis of the *kinB* region using Petrin [17] and MFOLD software [83] did not reveal any putative secondary structures characteristic of intrinsic terminators. Altogether, these observations suggest that the internal down-shift of *kinB* transcription is due to the activity of an intragenic Rho-dependent terminator.

We used a plasmid-based system for the detection of transcription terminators [116, 117] to more firmly establish the role of Rho in termination within the *kinB* gene. Two DNA fragments containing the *kinB* promoter, translation initiation region (TIR), and differently sized 5’-terminal regions of the *kinB orf* were cloned in front of the *cat* gene, encoding chloramphenicol (Cm) acetyltransferase (Fig 8B). The transcript of the long fragment (first 417 bp of *kinB orf*; plasmid pKinB-L*ong*) likely contained sequence features required for termination (estimated to be ~350 ribonucelotides downstream of the *kinB* start codon), whereas the transcript of the small fragment (first 157 bp of *kinB orf*; plasmid pKinB-S*hort*) did not (Fig 8B). The truncated *kinB orf* ended with a stop codon in both plasmids to ensure that translation of the *cat* mRNA initiating from the *kinB* promoter depended on its own RBS. At the same time, a transcription terminator located upstream of the *cat* gene would decrease *cat* expression and consequently Cm-resistance [117]. As shown in Fig 8C, BSB1 WT and RM cells containing the pKinB-S plasmid were mostly resistant to the tested Cm concentrations. In contrast, the pKinB-L plasmid conferred similar Cm-resistance only to RM cells, whereas WT cells containing this plasmid were considerably more sensitive (Fig 8C). These results are consistent with Rho-dependent termination within the large *kinB* fragment cloned into pKinB-L.

According to the *E*. *coli* model, Rho loads onto nascent transcripts not protected by translating ribosomes [22–25]. Therefore, Rho-dependent intragenic termination is modulated by the efficiency of translation initiation [118]. Thus, the efficiency of Rho would depend on *kinB* translation if it has a direct role in transcription termination within *kinB*. We varied the translation initiation rate of *kinB* by replacing its TIR, containing an imperfect RBS sequence (RBS wt), by a TIR with a strong RBS (RBSm^+^) or a TIR with degenerated RBS (RBSm^-^; Fig 8D) [119]. The pKinB-S-RBSm^+^ and pKinB-S-RBSm^-^ plasmids conferred similar levels of Cm-resistance in WT and RM cells as the pKinB-S plasmid (Fig 8E, data shown for WT cells). This demonstrated that modifications of the *kinB* TIR had no effect on translation of the *cat* mRNA. In contrast, WT cells carrying the pKinB-L-RBSm^+^ plasmid were considerably more resistant to Cm than cells carrying pKinB-L plasmid, whereas the absence of an active RBS in pKinB-L-RBSm^-^ resulted in much lower Cm-resistance (Fig 8F). However, *rho* inactivation in cells bearing the pKinB-L-RBSm- plasmid significantly improved their resistance to the antibiotic and restored viability at high Cm concentrations (Fig 8G).

Altogether, these results confirm the major role of Rho in the premature termination of *kinB* transcription.

### Rho-mediated control of *kinB* expression is reduced during sporulation

**Fig 9:**
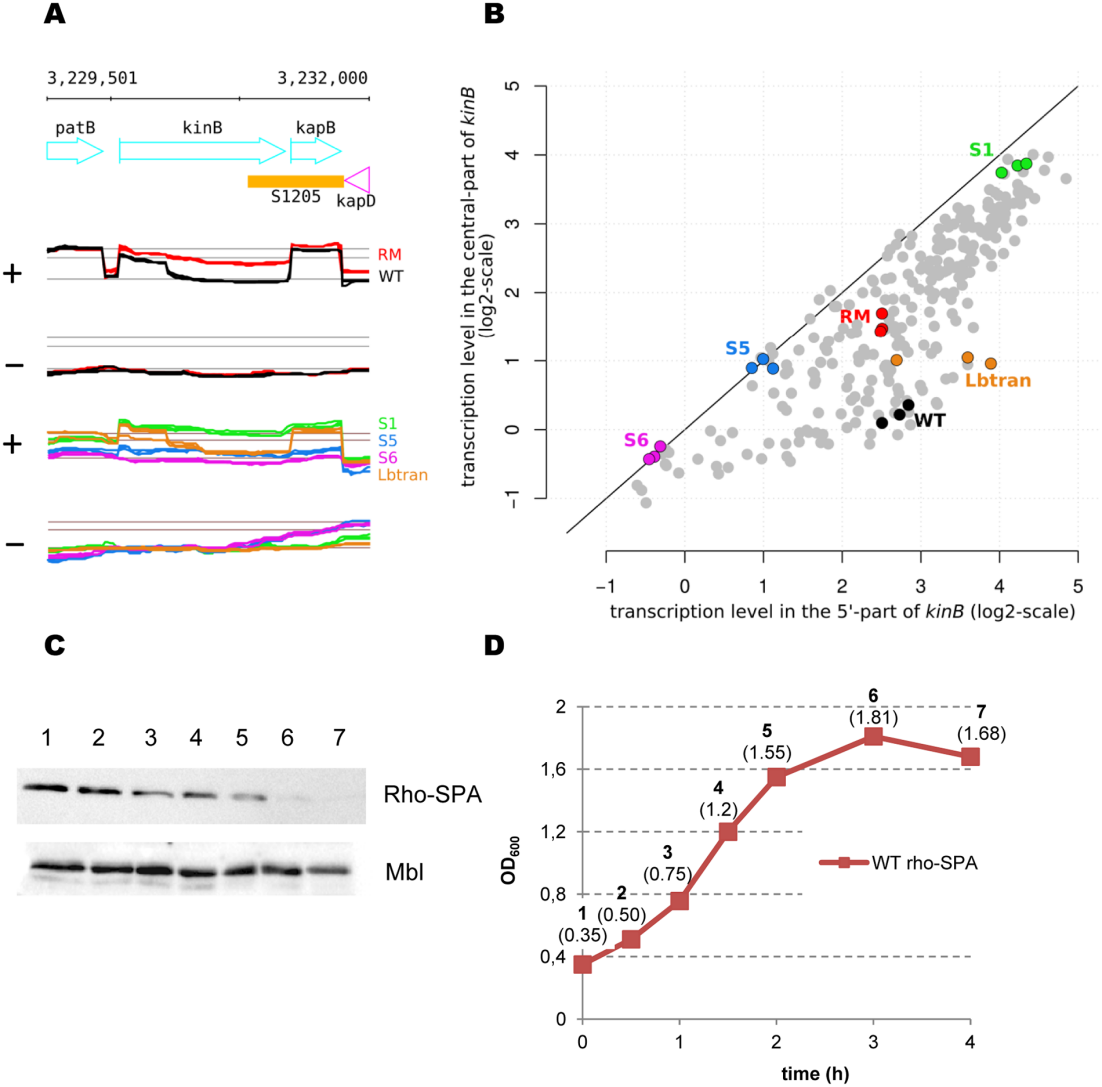
Rho-mediated control of *kinB* transcription is removed during sporulation. (A) The *kinB* gene is expressed differently during sporulation. Top and middle panels: Annotated genome and transcriptional profiles of *kinB* gene on the coding (+) and uncoding (−) strands in WT (black) and RM (red) cells during exponential growth in LB medium. Bottom panels: Transcriptional profiles of *kinB* gene in WT cells during transition phase (LBtran, orange), early (S1, green) and late (S5, blue and S6, pink) sporulation stages at both strands. The only normalization applied to the LBtran, S1, S5, and S6 profiles consisted in subtracting the chromosome median, whereas the RM and WT were subjected to the normalization procedure described in the Matherials and Methods section. The expression profiles are from [17] and include three biological replicas. (B) Comparison of expression levels between the 5’- and central parts of *kinB* gene across *B*. *subtilis* growth conditions represented by 269 re-annotated RNA samples collected for the wild-type [17]. The highlighted points correspond to the WT profiles shown in panel (A): LBtran (orange), S1 (green), S5 (blue), S6 (pink); and RM (red) and WT (black) during exponential growth in LB. Detailed values are provided in S5 Table. (C and D) Intracellular levels of Rho decrease during sporulation. *B*. *subtilis* BSB1 WT cells containing *rho*-SPA translational fusion at natural chromosomal locus were grown in sporulation- inducing DS medium at 37°C. Samples were taken at the indicated OD_600_ (D) and analyzed for Rho-SPA protein (C) as described in Materials and Methods. The three last samples (5 to 7) were taken from the sporulating cultures with a one-hour interval. To control equilibrium between the samples established by the Bradford analysis, total protein extracts were analyzed for Mbl protein using anti-Mbl specific antibodies. The experiment was reproduced three times. The results from the representative experiment are presented.

Thorough analysis of the *kinB* transcription profile in *B*. *subtilis* BSB1 across a database of 104 different growth conditions [17] revealed the presence of an intragenic down-shift of *kinB* expression in a substantial proportion of the dataset (S5 Table, Fig 9A and 9B). However, the expression level was remarkably similar between the 5’ and central segments of the *kinB* gene during the initial stages of sporulation, which correspond precisely to conditions in which *kinB* expression has been identified to be maximal (S5 Table). However, expression of the 5’ segment of *kinB* appeared to be relatively constant between non-sporulating cells (e.g. transition phase) and cells entering sporulation (e.g. S1; Fig 9B). This is in accordance with previous data, indicating that there is no specific regulation of the activity of the *kinB* promoter during sporulation [53]. Thus, intragenic transcription termination appears to be a mechanism for the control of *kinB* expression, which is weaker at the onset of sporulation.

The amount or activity of Rho should decrease during the early stages of sporulation if it regulates *kinB* expression. We assessed changes in the cellular level of Rho protein by constructing a Rho-SPA translational fusion, which retains regulatory activities of non-modified Rho (S8 Fig), and monitoring its expression in WT cells during growth in DS medium. Rho-SPA protein levels started to decrease after the mid-exponential growth phase and became barely detectable two hours after growth arrest (Fig 9C and 9D). This is in accordance with previous transcription analyses, showing that the level of *rho* expression is relatively high during exponential growth in rich medium and decreases during sporulation [17]. Previously, *B*. *subtilis* Rho was found to auto-regulate its expression by transcriptional attenuation at the Rho-dependent terminator(s) located within the leader region of *rho* mRNA [120]. Our results suggest that regulation of Rho expression during sporulation is more complex.

In conclusion, increased *kinB* expression due to the absence of intragenic transcription termination at early stages of sporulation appears to coincide with a decrease of Rho protein content. The mechanism(s) regulating Rho protein expression and/or stability during sporulation remain(s) to be elucidated.

### Rho overexpression inhibits sporulation and stimulates motility

The negative correlation between Rho content and *kinB* expression during sporulation in WT cells suggests that Rho termination activity may be dose-dependent. We tested this possibility by investigating the physiological effects of Rho overexpression using the middle-copy number plasmid pDG148Rho (hereafter pRho), which constitutively expresses Rho at a level which we estimate to be ~3-fold higher than normal (S9 Fig).

**Fig 10:**
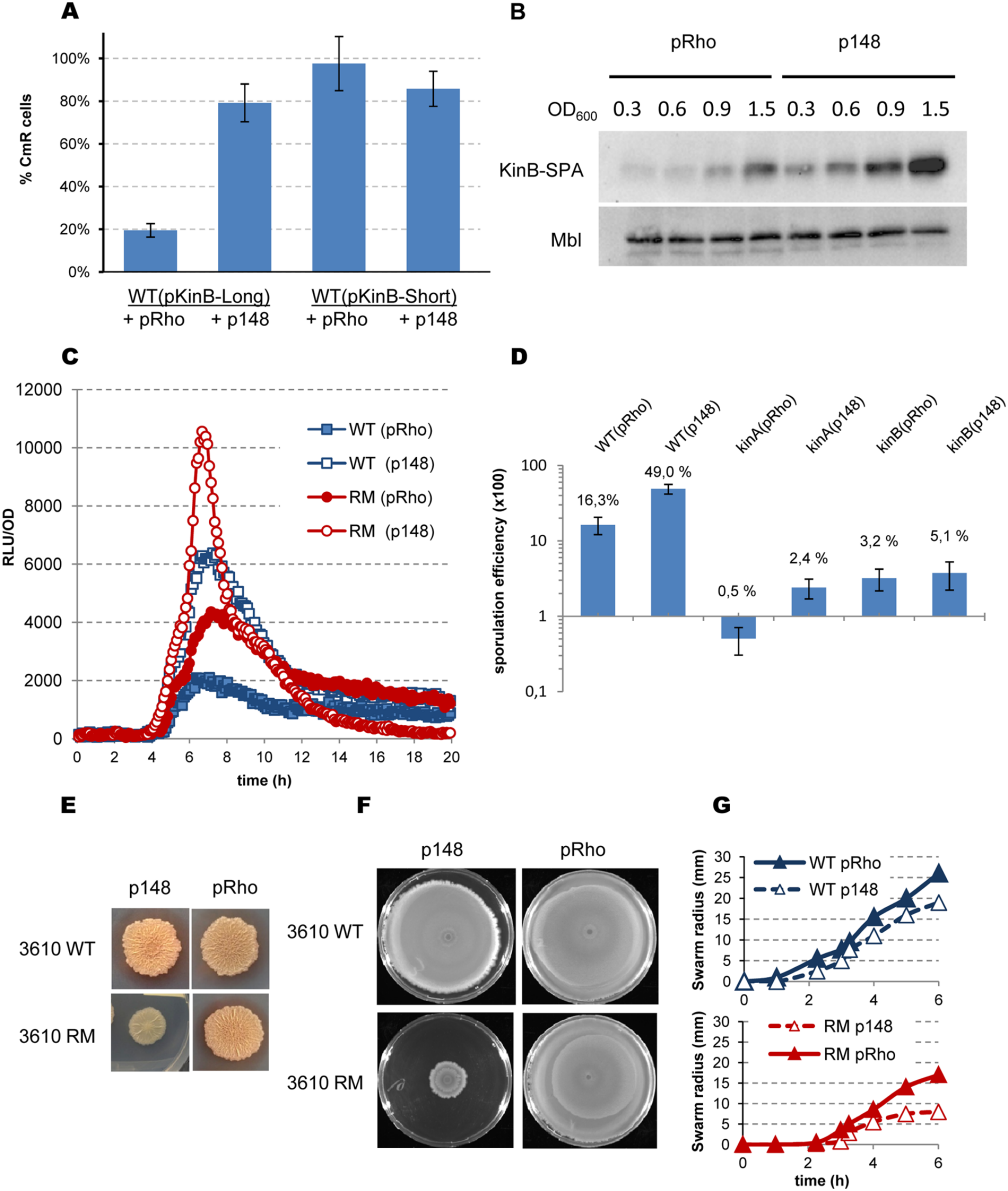
Effects of Rho over-expression in *B*. *subtilis* cells. (A) Over-expression of Rho reinforces Rho-dependent termination within *kinB* gene. The analysis of chloramphenicol-resistance of the BSB1 WT cells containing pKinB-L or pKinB-S plasmids described in Fig 8C was reproduced in the presence of Rho over-producing plasmid pRho or the control p148 vector; the applied concentration of Cm 3μg/ml. The experiment was repeated three times with nine biological replicas of each strain. (B) Over-expression of Rho suppresses synthesis of KinB in *B*. *subtilis* BSB1 WT cells. *B*. *subtilis* cells containing *kinB*-SPA translational fusion at natural chromosomal loci and pRho or p148 plasmids were grown in sporulation-inducing DS medium at 37°C to indicated OD_600_ and analyzed for KinB-SPA protein as described in Materials and Methods. To control equilibrium between the samples, total protein extracts were analyzed for Mbl protein using anti-Mbl specific antibodies. The experiment was reproduced twice. (C and D) Over-expression of Rho reduces sporulation. (C) Kinetics of luciferase expression from *spoIIA-luc* fusion in BSB1 WT (blue squares) and RM (red circles) cells in the presence of pRho (filled-in symbols) or p148 (opened symbols) plasmids. The experiments were performed as in Fig 6 three times. The results from the representative experiment are shown. (D) Analysis of the sporulation efficiency of *B*. *subtilis* BSB1 WT, BSB1 *kinA* and BSB1 *kinB* mutant cells containing pRho or p148 plasmids was performed as in Fig 7C after 20 hours of incubation in DS medium. Average values and standard deviations from three independent experiments are multiplied by 100 and plotted in a log10 scale. In total, twelve biological replicas of each strain were analyzed. (E) Colony biofilm formation by *B*. *subtilis* NCIB 3610 WT and RM cells containing pRho plasmid or p148 vector. Relevant genotypes are indicated on the side of each image. Each icon represents image of individual colonies grown on MSgg agar medium for 72h at 30°C. The experiment was reproduced at least five times. The results from the representative experiment are presented. (F and G) Over-expression of Rho reinforces cells motility. (F) Swarming motility of NCIB 3610 WT and NCIB 3610 RM containing pRho plasmid or p148 vector was assayed as in Fig 3 using LB 0.7% agar plates. The images were acquired after 20 hours of incubation at 37°C. The experiment was performed five times. The results from the representative experiment are shown. (G) Quantitative swarming assay of the NCIB 3610 (blue) and NCIB 3610 RM (red) strains containing pRho plasmid (solid lines; filled-in triangles) or p148 vector (dotted lines; empty triangles). Values represent the mean of five experiments including two replicas for each strain.

We started by testing the effect of Rho overproduction on the efficiency of the *kinB* intragenic Rho-dependent terminator. Introduction of the pRho plasmid into WT cells containing pKinB-L considerably decreased their Cm-resistance (Fig 10A, data shown for Cm 3μg/ml; compare with Fig 8C). In contrast, pRho did not affect Cm-resistance of WT cells carrying the pKinB-S plasmid, which does not contain the Rho-dependent terminator (Fig 10A). This indicated that the efficiency of the *kinB* intragenic transcription terminator increased with higher levels of Rho. Indeed, Western-blot analysis of the *kinB-SPA* translational fusion showed that cells grown in sporulation-inducing DS medium produced less KinB kinase in the presence of pRho than of a control vector (Fig 10B). Next, we assessed Spo0A phosphorylation and cell commitment to sporulation using the reporter *spoIIA-luc* fusion. Introduction of pRho into WT and RM cells decreased activity of the *spoIIA* promoter by ~3 fold relative to respective vector-containing controls (Fig 10C). These results suggest less efficient Spo0A phosphorylation when Rho is produced above its natural level. Of note, pRho-mediated inhibition of *spoIIA* activity was higher in the WT strain, which has the *rho* gene in the chromosome. The sporulation efficiency of the WT strain containing pRho was ~3 fold lower than that of control (Fig 10D), consistent with low Spo0A activation. A similar (~ 5-fold) inhibition of sporulation by pRho was observed in the BSB1 *kinA* mutant. In contrast, pRho had no significant effect on sporulation in BSB1 *kinB* cells, showing again that Rho preferentially targets *kinB* transcription (Fig 10D). Altogether, these results show that Rho overexpression inhibits sporulation, most likely by decreasing activity of the Spo0A phosphorelay system. One of the determinant factors of this inhibition is reinforced intragenic termination of *kinB* transcription.

Rho overexpression has the opposite effect of *rho* deletion, resulting in much less efficient sporulation. It is thus possible that Rho overexpression could stimulate the developmental programs for which Rho inactivation is inhibitory. We addressed this possibility by analyzing biofilm formation and swarming motility of strains containing pRho. Presence of the pRho plasmid rescued the negative biofilm phenotype of the NCIB 3610 RM strain, but had no global effect on naturally robust biofilms formed by NCIB 3610 WT (Fig 10E). Apparent insensitivity of biofilm formation to Rho overexpression in WT cells was expected due to known functional redundancy between phosphorelay kinases and the minor involvement of KinB in biofilm development under conditions of MSgg growth [85, 121]. However, the introduction of pRho not only suppressed the sessile phenotype of NCIB 3610 RM, but also increased swarming capacities of the parental swarming-proficient NCIB 3610 WT strain (Fig 10F and 10G). This higher-than-natural motility phenotype suggests that Rho over-production increases a subpopulation of WT cells with active SigD.

These experiments established that Rho inactivation and overexpression have opposite effects on *B*. *subtilis* physiology. This suggests that cells might be sensitive to intercellular variations of a naturally low level of Rho expression [120, 122].

## Discussion

Here, we report that pervasive transcription controlled by the termination factor Rho is an integrative element of the genomic regulatory networks that govern phenotypic heterogeneity and cell decision making in the Gram-positive model bacterium *B*. *subtilis*.

### Part of Rho-controlled pervasive transcription is relevant to cell differentiation

The transcriptome and proteome analyses presented here demonstrate the important effects of *rho* deletion on the *B*. *subtilis* gene expression landscape, encompassing approximately one-tenth of the known functional regions in a given growth condition. Prominent alterations of sense-strand expression in RM cells are caused by a combination of direct *cis* up-regulation due to transcription read-through of Rho-dependent terminators, and, in some cases, *cis* down-regulation of genes confronted with antisense transcription. These primary events propagate into regulatory networks and cause other changes that can be considered to be indirect *trans* effects. Recently, another transcription factor, NusA, was shown to regulate global gene expression in *B*. *subtilis* by controlling transcription read-through at suboptimal intrinsic terminators. Depletion of this essential protein caused a substantial increase of antisense transcription and misregulation of many genes mainly involved in DNA replication and DNA metabolism [84].

Physiological analyses of RM strains demonstrated that numerous changes in gene expression caused by Rho inactivation are not fortuitous. Indeed, they are related to biologically relevant phenotypes linked to three distinct *B*. *subtilis* cell fates: the synthesis of flagella leading to cell motility, matrix production underlying biofilm formation, and sporulation. These mutually exclusive developmental programs are controlled by complex regulatory networks, which are organized in a way that avoids their simultaneous activation in the same cell. Each program is characterized by a high level of phenotypic heterogeneity and bistable expression of a specific set of genes [37–40, 43, 44, 71]. The biological significance of Rho-controlled transcription is illustrated by the opposite phenotypes of RM and Rho over-expressing strains, corresponding to its high and low steady-state levels, respectively. RM cells are mostly sessile, exhibit low extracellular matrix production, and sporulate with high efficiency. In contrast, cells over-expressing Rho sporulate weakly, but are highly motile. These opposite phenotypes are determined by a specific architecture of the Rho-controlled transcriptome, of which the elements appear to be organized for the simultaneous stimulation of sporulation and repression of the principle alternative programs, once the control by Rho is removed.

### Rho-controlled transcription within motility and biofilm formation programs

Several Rho-controlled transcripts within the motility differentiation program act to down-regulate expression and activity of the motility-specific sigma factor SigD. In the absence of Rho, independent events of read-through transcription at Rho-dependent terminators directly up-regulate *slrR* and *slrA* genes which products negatively control *sigD* expression [59, 60–62, 71, 78]. Simultaneously, a Rho-dependent antisense transcript down-regulates the *flhO-flhP* operon, encoding components of the flagella export apparatus, which is essential for secretion of the anti-SigD factor FlgM [69, 81]. Altogether, these events disturb the self-reinforcing circuit of *sigD* expression and bias the SigD-ON/SigD-OFF bistable switch of motility towards a SigD-OFF state. Other factors contributing to motility are also affected by Rho inactivation, but were not analyzed in this study. For example, the flagellar chaperons FliS and FliD, encoded by the *yvyC-fliD-fliS-fliT* operon, were down-regulated in RM cells, probably as a result of the Rho-controlled asRNA targeting this locus (Table 1, S1 and S4 Tables).

Concomitant with the inhibition of flagellar synthesis, the absence of Rho severely alters the program of biofilm development. Both *epsA-epsO* and *tapA-sipW-tasA* operons encoding the main components of the extracellular matrix are down-regulated in RM cells. Weak expression of both matrix operons results from reinforcement of SinI/SinR-mediated repression of their promoters caused by a deregulated phosphorylation of Spo0A. Our analysis demonstrates that Spo0A~P rapidly accumulates beyond a level needed to induce matrix production and reaches a higher, sporulation-triggering, threshold, due to increased activity of Spo0A phosphorelay in RM cells. High Spo0A~P should inhibit expression of the SinI anti-repressor and, consequently, reinforce SinR-mediated repression of the matrix operons.

Besides this indirect *trans* effect on the expression of matrix operons, inactivation of Rho provokes the generation of a new antisense transcript, which spans across the entire *epsA-epsO* operon and, as shown here, contributes to the inhibition of biofilm formation in RM cells. The exact mechanism by which *eps* asRNA inhibits expression of the *eps* operon remains to be established, but it may directly interfere with activity of the *eps* promoter and/or the RNA switch located between the *epsB* and *epsC* genes, which allows transcription of the long *eps* operon [123], or act post-transcriptionally [30].

Additionally, we detected down-regulation of the *skf* operon (S1 Table; http://genome.jouy.inra.fr/cgi-bin/seb/index.py) involved in the production and release of the cannibalism toxin, Skf, known to stimulate biofilm development and delay sporulation [38, 124]. Thus, inefficient production of Skf might have also contributed to reduced biofilm formation by RM cells. Expression of the *skfA-H* operon is inhibited by a high level of Spo0A~P [43, 58], similarly to the matrix operons. In addition, highly expressed asRNA overlapping the entire *skf* operon was revealed in the RM strains (S1 Table). The putative role of the *skf*-specific asRNA in the regulation of the *skfA-H* operon merits further analysis.

### Rho-controlled transcription within the Spo0A phosphorelay

All Rho-controlled transcripts detected within the Spo0A phosphorelay are sense and lead to up-regulation of the cognate genes when de-repressed in the absence of Rho. Aside from the *rapA* gene, other targets of Rho-controlled transcription encode positive factors of Spo0A phosphorylation: sensor kinase KinB, its positive effector KbaA, and the phosphotransferase Spo0B. Pervasive transcription, together with several positive feedback loops of *spo0A* expression, intensifies phosphorelay activity. As a result, RM cells engage in differentiation more efficiently and synchronously than WT cells, characterized by broadly heterogeneous Spo0A phosphorylation [104].

Key factors involved in the increase of phosphorelay activity in RM cells are the up-regulated KinB and, to a lesser extent, KinA kinases, which are known to trigger sporulation if over-expressed [49, 63]. In RM cells, *kinA* up-regulation is most likely due to increased Spo0A~P, acting within a positive auto-regulatory loop [104], and thus would be an indirect *trans* effect of Rho-controlled transcription. In contrast, up-regulation of *kinB* in RM cells is the direct result of a read-through at intragenic transcription terminator. We experimentally proved that the 5’-terminal segment of the *kinB* transcript contains sequence features required for Rho-dependent transcription termination. We showed that the efficiency of intragenic *kinB* termination depends on Rho availability and negatively correlates with the *kinB* translation initiation rate in a Rho-dependent manner. This anti-correlation is in accordance with the well-documented preferential activity of Rho at untranslated RNAs and its role in the control of transcription-translation coupling [23–25, 118].

The global transcriptional regulators, AbrB and CodY, and positive stringent control regulate the expression of KinB at the transcription initiation level [125–127]. Our results highlight a novel regulatory mechanism of *kinB* expression that acts through the premature termination of transcription. Inactivation of Rho and, consequently the lack of termination, generates a full-length *kinB* transcript, leading to an increased level of KinB, higher levels of Spo0A~P, and the activation of sporulation. In contrast, high Rho amounts strengthen intragenic termination of *kinB*, decrease cellular levels of KinB and phosphorelay activity, and lead to a weak-sporulation phenotype.

The intragenic termination of *kinB* in WT cells is less efficient at early stages of sporulation, correlating with decreased *rho* expression. Programmed decrease of Rho amount might be needed to compensate probable strengthening of the premature *kinB* termination due to decrease of the translation efficiency under nutrient-limiting conditions. This emphasizes the biological significance of Rho-mediated control of *kinB* expression. By premature termination of *kinB* transcription, Rho delays sporulation, giving cells the possibility to continue exploring the environment. Rho might also influence other developmental programs, as KinB kinase has been found to be involved in the control of sliding motility and biofilm formation under particular growth conditions [47, 128, 129]. Thus, Rho-mediated control of *kinB* expression appears to be an integral part of the deterministic regulation of *B*. *subtilis* development, mediated by Spo0A.

Recent study in *E*. *coli* has established that Rho acts as a global regulator of genes expression during the exponential growth by prematurely terminating transcription within the 5’ un-translated regions of hundreds of genes, including the global stress response sigma factor *rpoS* gene. Rho-mediated control of *rpoS* expression is modulated by the regulatory sRNAs and is relieved at the stationary phase of growth [130]. This and our analyses illustrate the diversity of strategies by which bacteria employ Rho-mediated transcription termination to adapt to the environmental and metabolic changes.

### Rho as a factor of heterogeneity in *B*. *subtilis*

Our data suggest another potential role of Rho: that as a factor of phenotypic heterogeneity within *B*. *subtilis* population. Heterogeneity based on intrinsic fluctuations of gene expression provides potential flexibility to a genetically homogenous population to respond to environmental changes [131]. We showed that population heterogeneity is considerably reduced when Rho-controlled transcription levels are artificially high or low. This suggests that pervasive transcription in WT cells varies depending on the intracellular concentration of Rho. This was already suggested by our previous study, which noted that the length of sense and antisense 3’extensions generated by read-through transcription negatively correlates with *rho* expression [17].

*B*. *subtilis* Rho is a low abundant protein present at 0.5 to 4.8% of the level of RNA polymerase [17, 120, 122]. According to the *E*. *coli* paradigm, Rho should function as a hexamer [22]. This suggests that even minor variations of Rho levels might have substantial impact on the efficiency of pervasive transcription. The stochastic generation of pervasive, often antisense, transcription targeting gene expression via various mechanisms [29, 30, 132] may increase intercellular heterogeneity and phenotypic variation within isogenic *B*. *subtilis* population. A similar hypothesis was recently proposed following analysis of the *Clostridium botulinum* Rho protein expressed in heterologous systems [133].

Comparative analyses of closely related bacterial species have revealed both conservation of and significant differences between respective non-coding transcriptomes. It has thus been proposed that pervasive transcription represents a major element of inter-strain divergence, providing a potential for physiological adaptation [8, 134, 135]. Here we show that several transcripts regulated by Rho are similarly functional in different *B*. *subtilis* strains (BSB1, NCIB 3610, PY79). Moreover, the nucleotide sequences of the corresponding genomic regions are conserved among *B*. *subtilis* strains (≥ 99% identity), while the conservation level is lower in other *Bacillus* species (for example, the *kinB* and *flhO-flhP* genomic regions of *B*. *subtilis* and *B*. *amyloliquefaciens* present only 67%-73% of nucleotide identity, respectively). Thus, RNA features controlled by Rho might represent a specific trait fixed by evolution, at least in *B*. *subtilis*.

It is important to note that the Rho-dependent regulatory network in *B*. *subtilis* may be broader than it emerges from our analysis, as Rho-controlled transcripts expressed under other conditions and/or dependent on alternative sigma factors, may have escaped identification. Future studies in *B*. *subtilis* and other bacteria will help to understand how elements of the Rho-controlled pervasive transcription are recruited to achieve important regulatory functions.

### Concluding remarks

Our results support the view that, in terms of gene regulation, transcription termination can be as important as the repression or activation of transcription initiation. They advance our understanding of the role of pervasive transcription in bacteria, considered for a long time as a “dark matter” of bacterial transcriptomes. Part of Rho-controlled transcription appears to constitute an integral module of the *B*. *subtilis* cell differentiation regulatory network instead of simply being non-functional transcriptional noise. This ranks termination factor Rho among the global regulators of *B*. *subtilis*.

## Materials and methods

### Bacterial strains and growth conditions

*B*. *subtilis* strains used in the work are listed in S6 Table. When needed, cells contained plasmids as indicated in Results section. Cells were routinely grown in Luria-Bertani liquid or solidified (1.5% agar; Difco) medium at 37°C. Standard protocols were used for transformation of *E*.*coli* and *B*. *subtilis* competent cells. SPP1 phage was used for transduction of NCIB 3610 strains as described [136]. Biofilm formation was analyzed in liquid (for pellicles) or solid (for colony biofilms) MSgg medium [96]. Sporulation was analyzed in supplemented Difco Sporulation medium (Difco) [137]. When required for selection, antibiotics were added at following concentrations: 100 *μ*g per ml of ampicillin, 60 *μ*g per ml of spectinomycin, 0.5 *μ*g per ml (for *B*. *subtilis*) and 90 μg per ml (for *E*. *coli*) of erythromycin, 3 μg per ml of phleomycin, 5 μg per ml of kanamycin, and 5 *μ*g per ml of chloramphenicol.

### Strains and plasmid construction

*B*. *subtilis* strains and the plasmids used in the study are listed in S6 Table. The plasmids were constructed in *E*. *coli* TG1 strain. The used oligonucleotides are listed in S7 Table.

To repair NCIB 3610 RM *(*Δ*rho*::*phleo*) strain back to the wild type *rho* allele, a near-by DNA fragment containing *ywjH* gene was amplified using oligonucleotides eb460 and eb461, digested by HindIII and BamHI endonucleases and cloned at pMutin4 plasmid [138]. The resulting plasmid was integrated by single crossover in the *ywjH* locus of BSB1 chromosome and subsequently transferred in NCIB 3610 RM with selection at erythromycin. NCIB 3610 RM *ywjH*::*pMutin* transductants were tested for sensitivity to phleomycin that indicated substitution of the *rho* deletion by the wild type *rho* allele. Phleomycin-sensitive clones were further selected for loss of the erythromycin-resistance that indicated excision of pMutin4 from the chromosome and restoration of the *ywjH* gene. Thus obtained NCIB 3610 *rho* wt* clones were controlled for integrity of the *rho* and *ywjH* wild type alleles by PCR.

To inactivate *kinB* gene, the internal part of the gene was amplified using oligonucleotides veb608 and veb610, digested with HindIII and EcoRI endonucleases and cloned at pMutin4 plasmid. The resulting plasmid was integrated by single crossover in the *kinB* locus of BSB1 chromosome leading to its disruption.

The translational fusions of the *kinA*, *kinB* and *rho* genes with the sequential peptide affinity (SPA) tag sequence were constructed for immunoblot analysis of the proteins. The 3’- terminal parts of the *kinA* and *kinB* genes were amplified using pairs of oligonucleotides eb625 and eb626 and veb606 and veb607, respectively. The amplified *kinA* and *kinB* DNA fragments were digested, respectively, by BamHI and NcoI and by Acc65I and NcoI endonucleases, and ligated with pMutin-SPA plasmid [139] cutted by BglII and NcoI for the *kinA*, and by Acc65I and NcoI for the *kinB* cloning. The *rho-SPA* fusion was constructed using ligation-independent cloning as described [140]. The 3’-terminal part of *rho* was amplified using oligonucleotides rhoSpa-Fwd and rhoSpa-Rev, treated with T4 DNA polymerase in the presence of dTTP and annealed to pMUTIN-LICSPA plasmid [141], linearized by AscI endonuclease and treated with T4 DNA polymerase in the presence of dATP. The annealing mixture was transformed into *E*.*coli* cells. The resulting plasmids with the *kinA-*, *kinB-* and *rho-SPA* fusions were transferred in BSB1 cells where they integrated into respective chromosomal loci by single crossover.

To express the *flhO* and *flhP* genes from ectopic position, the *flhOP* operon was PCR-amplified using oligonucleotides eb617 and eb618, digested by Acc65I and BamHI endonucleases and cloned onto integrative plasmid pSG1729 [142]. The resulting plasmid was integrated in the *amyE* locus of BSB1 chromosome by double crossover with selection of the chloramphenicol-resistant transformants, which lost amylase activity.

To suppress anti-sense transcription within the *eps* operon, the 5’-part of *epsO* gene was amplified using oligonucleotides veb680 and veb681, cutted by EcoRI endonuclease and cloned at pMutin4 plasmid between PacI site filled-in by T4 polymerase and EcoRI site. In the resulting plasmid, the 3’ end of the inserted fragment is flanked by three intrinsic transcription terminators of the vector [138]. The plasmid was integrated in the *epsO* locus of BSB1 chromosome by single crossover and subsequently transferred in NCIB 3610 WT and RM cells. In the mutant *epsO*:*Ter* allele, the inserted transcription terminators are oriented to block transcription of the *eps* asRNA initiated near the 3’- end of *epsO* gene.

To analyze genes expression, pUC18Cm-luc plasmid was used to construct genes transcriptional fusions with the butterfly luciferase gene *luc* [97]. The promoters of *spoIIA*, *gerE*, *epsA* and *tapA* genes were amplified together with the upstream chromosomal fragments of ~1 Kbp, using the corresponding pairs of oligonucleotides listed in S7 Table. The fragments containing *spoIIA* and *gerE* promoters were digested by HindIII and BamHI endonucleases and cloned at pUC18Cm-luc. The *luc* fusions with *epsA* and *tapA* promoters were obtained by the assembly Gibson’s procedure with linear vector amplified with oligonucleotides F- pUC18-luc and R- pUC18-luc [143] (S7 Table). The obtained plasmids were used to transform *B*. *subtilis* where they integrated by single crossover at chromosomal loci of the targeted genes. This event reconstructs natural regulatory region of gene upstream the fusion and an intact copy of gene downstream.

To inactivate the anti-SigD factor FlgM without inducing polar effects on the downstream genes, a marker-less in-frame *flgM* deletion was constructed by a two-step integration-excision method similar to the previously described [144]. Two chromosomal fragments of ~1 Kbp were amplified using oppositely oriented and partially over-lapping oligonucleotides veb687 and veb688, which match close to the extremities of *flgM* gene, and their counterpart primers veb686 and veb689, respectively. The amplified fragments were joined by PCR using primers veb686 and veb689 and cloned between the BamHI and SalI sites at the thermo-sensitive plasmid pMAD [145]. The resulting plasmid contains a fragment of *B*. *subtilis* chromosome with in-frame deletion of the most part of *flgM* gene (*flgM*Δ*63*). The *flgM*Δ*63* structure was controlled by sequencing. The plasmid was transformed in BSB1 cells with selection for erythromycin-resistance at non-permissive for replication temperature 37°C. This led to plasmid insertion into the chromosome by single crossover and duplication of *yvyF-csrA* locus, which copies contained the wild type or *flgM*Δ*63* alleles. The duplicated region was transferred in NCIB 3610 RM cells, which were further cultivated at permissive 30°C without erythromycin to stimulate excision of the *yvyF-csrA* duplicate from the chromosome. The resulting clones were controlled for the presence of *flgM*Δ63 allele by PCR using primers veb686 and veb689.

For Rho overproduction, *rho* gene was amplified using oligonucleotides eb423 and eb424; digested by NheI and BamHI endonucleases and cloned at pDG148 [146] plasmid between the XbaI and BamHI sites. The resulting plasmid pRho is deleted for *lacI* repressor gene and expresses Rho constitutively from Pspac promoter. To construct the control vector pDG148Δlac, the XbaI-BamHI double-cutted pDG148 was treated by T4 DNA polymerase and self-ligated. To estimate Rho production from P_spac_ promoter, pRho plasmid was modified to express a SPA-tagged protein. The 3’-terminal part of the chromosomal *rho-SPA* fusion (see above) was amplified using oligonucleotides eb423 and pdg148-rev, which matches the sequence behind the SPA-tag, and digested by XhoI endonuclease. The PCR product was cloned at pRho plasmid between BamHI site filled-in by T4 DNA polymerase and XhoI site.

To analyze presence of the Rho-dependent terminator within *kinB* gene, two plasmids were constructed which contain *kinB* promoter and differently sized 5’-terminal parts of the gene. The *kinB* gene and the upstream 650 bp region were amplified using oligonucleotides veb610 and veb611. The fragment was digested either by SmaI and NsiI or by SmaI and MboI endonucleases and the fragments of 390 and 650 bp, respectively, were gel-purified and cloned at the terminator-screening vector pGKV210 [116] digested, respectively, by SmaI and PstI or by SmaI and BamHI endonucleases. The resulting plasmids pKinB-S(*hort*) and pKinB-L(*ong*) were transformed into *B*. *subtilis* cells with selection at erythromycin. Both plasmids contain the promoter of *kinB* gene and the first 157 and 417 bp of *kinB orf*, respectively.

To modify efficiency of *kinB* translation initiation at pKinB-L and pKinB-S plasmids, the whole pKinB-S was amplified using the oligonucleotides veb678 and veb679 or veb679 and veb690 designed to substitute natural *kinB* ribosome binding site (RBS) by a stronger or a weaker, respectively. PCR products were treated by DpnI endonuclease, to degrade template DNA, 5’-phosphorylated by T4 polynucleotide kinase, self-ligated and transformed in *E*. *coli* cells with selection at erythromycin. The resulting plasmids were used as templates for amplification of the modified *kinB* fragments using the oligonuclotides veb676 and eb407. PCR products were controlled by sequencing, digested by EcoRI and NarI endonucleases and cloned at similarly digested pKinB-L or pKinB-S. The resulting derivative plasmids contain either canonic GGAGGA (RBSm^+^) or a weak TGATAA (RBSm^-^) RBSs (Fig 8D).

### Tiling array data analysis

Tiling array data obtained with a strand-specific resolution of 22 bp for exponential growth in LB [17] were reanalyzed. This re-analysis used the same signal processing and gene-level aggregation procedures as the initial study but differed by the normalization and differential expression analysis. Briefly, the raw log2-transformed hybridization signal was smoothed with an algorithm that accounts for probe-specific biases and changes in expression levels between adjacent regions that can take a form of abrupt shifts and more continuous drifts [17]. Then, a whole genome transcription profile was aggregated into sense and antisense gene level data by computing the median of the smoothed signal on the sense and antisense strand of a repertoire of native expression segments, *i*.*e*. detected as transcribed in the wild-type in one out of 269 hybridized RNA samples intended to capture the diversity of the lifestyles of the bacterium [17].

To allow precise between-sample comparison of expression levels on the both sense and antisense strands of the native expression segments, the quantile normalizing transformation fitted on aggregated sense strand levels was also applied to the antisense strand levels and to the smoothed transcription profiles as described [20]. Statistical comparison of the 3 biological replicates for RM and WT relied on moderated t-statistics computed with the functions “lmFit” and “eBayes” of R package “limma” [147]. Control the False Discovery Rate relied on q-values obtained with R package “fdrtool” [148]. Sense strand and antisense strand levels were considered simultaneously in these analyses.

The same statistical procedure served here to examined the expression levels immediately downstream a repertoire of 3242 transcriptional up-shifts encompassing promoters of most genes [17]. We considered that an up-regulation was detected at a given promoter if the downstream smoothed-normalized signal exhibited differential expression according to the specified amplitude (log2 RM/WT ≥ 1) and false discovery rate (q-value ≤ 0.01) cut-offs and if the downstream transcription level was at least twice higher than the upstream level in the 3 biological replicates for RM (indicating activity of this promoter as opposed to transcription from an upstream promoter). Reciprocally, down-regulation was considered detected when log2 RM/WT ≤ -1, q-value ≤ 0.01, and the downstream transcription level was at least twice higher than the upstream level in the 3 biological replicates for WT.

### RNA extraction and RT-PCR

RNA was extracted from *B*. *subtilis* WT and RM derivative strains grown in LB or MSgg medium at 37°C under vigorous agitation up to an OD_600nm_ ~0.5. RNA preparation and DNase treatment were done as described [17]. Quality and quantity of RNA samples were analyzed on Bioanalyzer (Agilent, CA). For analysis of the antisense transcription of *flhOP* and *eps* operons by RT-PCR, cDNA was synthesized using *flhO*, *epsL* and 16S rRNA specific oligonucleotides eb700, eb706 and eb715, respectively, (S7 Table) and 50 ng of total RNA as a template in reaction with SuperScriptIV Reverse Transcriptase (Invitrogen) according to the supplied protocol, and treated with RNaseH (Invitrogen) for 20 min at 37°C. To amplify internal DNA fragments, PCR (35 cycles) was performed by Thermo Scientific DreamTaq DNA Polymerase (ThermoFicher) with the oligonucleotides pairs specific for *flhO* (eb705 and eb702), *eps*K (eb708 and eb710) and 16S rRNA genes (eb715 and eb716) (S7 Table).

### RNA seq library preparation and data analysis

RNA samples of *B*. *subtilis* BSB1, NCIB3610, and the corresponding *rho*-deletion mutants were prepared as described in the previous section. Transcriptome analysis was performed by Transcriptome and EpiGenome platform (Pasteur Institute, France). Briefly, RNA samples have been submitted first to ribosomal RNA depletion using the RIBOZero rRNA removal kit Bacteria (Illumina, San Diego, California). Purified fraction was then treated for library preparation using the Truseq Stranded mRNA sample preparation kit (Illumina, San Diego, California) according to manufacturer’s instruction. Fragmented RNA samples were randomly primed for reverse transcription followed by second-strand synthesis to create double-stranded cDNA fragments. No end repair step was necessary. An adenine was added to the 3'-end and specific Illumina adapters were ligated. Ligation products were submitted to PCR amplification. Sequencing was performed on the Illumina Hiseq2500 platform to generate single-end 65 bp reads bearing strand specificity. Reads were trimmed based on sequencing quality using Sickle (v1.200) and mapped on AL009126.3 reference genome assembly using Bowtie2 (2.2.6; options "-N 1 -L 16 -R 4") [149] before read-count aggregation on the sense and antisense strand of each transcribed region (annotated genes and S-segments) with Htseq-count (0.6.0; standard options). Raw sequencing data and aggregated counts have been deposited in GEO (GEO submission number GSE94303). Experiments were made in duplicates for *B*. *subtilis* NCIB3610 to allow statistical differential expression analysis. RPKM normalization [150] served for a first level of exploratory analysis. Differential expression analysis of *B*. *subtilis* NCIB3610 relied on R library “DESeq2” [151] and associated "median ratio method" normalization procedure. Normalization relied on a control set of 1152 always well expressed sense regions a priori less impacted by low-level transcriptional read-through typical of the rho-deletion mutants. These were ranking in the 25% highest density of mapped reads in each of the four NCIB3610 samples. DESeq2 p-values were converted into q-values using R library “fdrtool” [148]. While the initial differential expression analysis relied on the four NCIB3610 samples, we also performed another, more discriminative, differential expression analysis excluding one of the parental NCIB3610 sample which exhibited anti-sense transcription levels markedly higher than other RNA-Seq (*B*. *subtilis* BSB1 and NCIB3610) and tiling array (*B*. *subtilis* 1012) samples of the parental strains probably because of more advanced growth status.

### Preparation of protein samples and LC-MS/MS analysis

*B*. *subtilis* 168 derivative strains were grown in LB medium at 37°C under vigorous agitation up to an OD_600nm_ ~0.6. Cells were harvested by centrifugation (6,000g for 10 min at 4°C), washed once with 50 mL of buffer A (10 mM Tris-Cl pH 7.5, 150 mM NaCl) before being centrifuged again. The cell pellets were frozen in liquid nitrogen and kept at -80°C. Cell pellets were thawed on ice and resuspended with 5 mL of buffer A, and disrupted by French press (pressure 2.7 MPa). Unbroken cells were removed by centrifugation at 15,000 RPM, and the supernatants were centrifuged at 100,000g for 1 hour at 4°C. The resulting supernatants were kept as the cytosolic fraction. The pellets were then washed twice with cold buffer A, and centrifuged twice at 100,000g for 1 hour at 4°C. The pellets were re-suspended in TE (20mM Tris, 2 mM EDTA) and considered as the membrane fraction. All experiments were carried out in duplicate. Membrane and cytosolic protein concentrations were measured using the Bradford method (Bio-Rad kit). Membrane and cytosolic samples were treated differently before separation by electrophoresis. Samples corresponding to the membrane fractions were mixed with a loading buffer containing 125 mM Tris-Cl pH 6.8, 20% glycerol, 10% SDS and 0.1% bromophenol blue, and left overnight at room temperature. Equal amounts of cytosolic proteins for each sample were treated with a classic Laemmli loading buffer and boiled for 5 min. Samples were then loaded on a 10% Bis-Tris polyacrylamide NuPAGE gel (Invitrogen) and the electrophoresis was left running at 100V for 15 min. The gel was then stained with Bio-Safe Coomassie G-250 Stain (Bio-Rad). After distaining the bands of 2 mm-wide along the protein migration lane were cut off and used as samples for the identification of the proteins by mass spectrometry. The gel pieces for each sample were washed twice with 0.2% TFA-50% acetonitrile, reduced by 10 mM DTT for an hour at 56°C, alkylated by 50 mM iodoacetamide for 1 hour at room temperature into darkness. Sequencing grade modified trypsin (Promega) diluted in 25 mM NH_4_HCO_3_ was added for 18 hours at 37°C. Tryptic peptides were recovered by washing the gel pieces twice with 0.2% TFA-50% acetonitrile, once with 100% acetonitrile and the supernatants were evaporated to dryness. The peptides were then re-suspended in 25 μL of pre-column loading buffer (0.05% trifluoroacetic acid (TFA) and 5% acetonitrile (ACN) in H_2_O), prior to LC-MS/MS analysis. Mass spectrometry was performed on the PAPPSO platform (MICALIS, INRA, France, http://pappso.inra.fr/). Protein identification was performed with X!Tandem software (DB: X!tandem version 2013.09.01.1) against a protein database of *B*. *subtilis* as well as a proteomic contaminant database (for details of the parameters used, see S2 Table). For quantification of the proteins, we used the number of spectra obtained during protein identification by mass spectrometry. The number of spectra is admitted to be proportional to the abundance of a given protein. For each protein, we calculated the relative abundance factor (PAI) as described in [66]. The PAI estimates the relative abundance of a protein and is calculated as the number of identified spectra divided by the number of theoretical peptides of the protein (theoretical peptide number corresponds to the number of peptides resulting from the theoretical digestion of the protein by trypsin and that are visible in mass spectrometry [i.e. having a mass ranging between 800 and 2,500 D.]). The PAI were log2-transformed after adding a pseudo count of 0.1 which corresponded approximately to quantile 10% of the PAI distributions.

### Luciferase assay

Analysis of the promoters’ activity using translational fusions with luciferase was performed as described by [97] with minor modifications. Cells were grown in LB medium to mid-exponential phase (optical density OD_600_ 0,4–0,5 with NovaspecII Visible Spectrophotometer, Pharmacia Biotech), after which cultures were centrifuged and resuspended in fresh DS or MSgg media to obtain OD_600_ 1,0. The pre-cultures were next diluted in respective media to OD_600_ 0.025. The starter cultures were distributed by 200μl in a 96-well black plate (Corning) and D-lucefirin (PerkinElmer) was added to each well to final concentration 1.5 mg/mL. The cultures were incubated with agitation at 37°C in PerkinElmer Envision 2104 Multilabel Reader (PerkinElmer) equipped with an enhanced sensitivity photomultiplier for luminometry (data presented in Figs 4, 6 and 10) or in Synergy 2 Multi-mode microplate reader (BioTek Instruments; data presented in Fig 7 and S7 Fig). Relative Luminescence Units (RLU) and OD_600_ were measured at 5 min intervals. Each fusion-containing strain was analyzed at least three times. Each experiment included four independent cultures of each strain.

### Motility assays

Swarming and swimming motility tests were performed using NCIB 3610 strain and its derivatives as described by [68, 152] with some modifications. The fresh plates (9cm) were prepared from liquid LB medium (Difco) fortified by agar (Invitrogen, Life Technologies) at 0.3% or 0.7% concentration for swimming and swarming tests, respectively, and dried in a laminar flow hood for 15 minutes. *B*. *subtilis* cells were grown to an OD_600_ 0.5 (Biochrom Libra S11 Visible Spectrophotometter, Biochrom), 2 ml of cells were pelleted and gently resuspended in 100 μl of phosphate-buffered saline (137 mM NaCl, 2.7 mM KCl, 10 mM Na_2_HPO_4_, 2 mM KH_2_PO_4_) containing 0.5% of India ink (Higgins). For motility assay, 5μl of cells were directly spotted on the plate and dried in a laminar flow hood for two minutes. Plates were incubated at 37°C and the extent of swimming or swarming was noted at defined time intervals. The images were acquired with the ChemiDoc MP system (BioRad) and treated using ImageLab 5.0 software (BioRad) after 5 or 20 hours of incubation for swimming or swarming motility tests, respectively. For each strain, from three to five independent cultures were analyzed in parallel during each experiment. At least four independent experiments were performed.

### Colony and pellicle biofilm formation assays

Overnight bacterial cultures were diluted 200-times in fresh LB medium and grown at 37°C with agitation to an OD_600_ ∼0.6. For the colony assay, 2μl of culture was spotted onto MSgg agar plate (1.5% agar, Invitrogen) and incubated at 30°C for 72h. For pellicle assay, 2μl of culture was added to 2ml of MSgg medium in a well of 24-well sterile microtiter plate (Evergreen Scientific). The plates were incubated without agitation at 30°C for 72h. Photographs were acquired with the Samsung Galaxy Tab E–SM-T560. For each strain, four independent cultures were analyzed in parallel during each experiment. At least five independent experiments were performed.

### Sporulation assay

For sporulation assay, cells were diluted in LB in a way to obtain the exponentially growing cultures after over-night incubation at 28°C. The pre-cultures were diluted in pre-warmed liquid DS medium at OD600 0.025 and incubated at 37°C. The growth rates were the same for all strains. Starting from OD_600_ 1.5 (taken as T0) cultures were analyzed for the presence of spores at the indicated time. Samples were split in two and one part was heated at 75°C for 15 min; heated and unheated cultures were plated in sequential dilutions at LB-agar plates and incubated for 36 h at 37°C. The percentage of spores was calculated as the ratio of colony forming units in heated and unheated samples. Each experiment included three independent isogenic cultures. Four independent experiments were performed to establish sporulation efficiency of each strain.

### Western blotting

The crude cell extracts were prepared using Vibracell 72408 sonicator (Bioblock scientific). Bradford assay was used to determine total protein concentration in each extract. Equal amounts of total proteins were separated by SDS-PAGE (12% polyacrylamide). The SPA-tagged Rho, KinA and KinB proteins were visualized using the primary mouse ANTI-FLAG M2 monoclonal antibodies (Sigma-Aldrich; dilution 1:5,000) and the secondary goat peroxidase-coupled anti-mouse IgG antibodies (Sigma-Aldrich; dilution 1:20,000). MreB and Mbl proteins used as controls for samples equilibrium were visualized using primary rat anti-MreB and rabbit anti-Mbl antibodies (a gift of X. Henry, dilution 1:10,000) and the secondary peroxidase-coupled anti-rat and anti-rabbit antibodies A9037 and A0545, respectively (Sigma-Aldrich; dilution 1:10,000). Three independent experiments were performed, and a representative result is shown.

## Supporting information

S1 Fig RT-PCR analysis of antisense transcription of *flhOP* operon in the BSB1 and NCIB 3610 WT and RM strains. Total RNA was extracted as described in Materials and Methods and cDNA synthesis was performed using 50 ng of total RNA as template and mixture of specific oligonucleotides. Reactions were performed with (+) and without (−) reverse transcriptase (RT). PCR was done with oligonucleotides specific for *flhO* asRNA (asRNA *flhO*, top section) and for rRNA (rRNA, bottom section) (S5 Table).
(TIF)

S2 Fig Extension of S1475 RNA in the 1012 RM strain. Genomic region corresponding to the *slrA* gene shows the extension of S1475 RNA (can be visualized on http://genome.jouy.inra.fr/cgi-bin/seb/index.py). Sections show annotated genome (top) and expression profiles of WT (black) and RM (red) on the (+) and (–) strands (mid and bottom sections).
(TIF)

S3 Fig Deletion of the *flgM* gene alleviates motility defect of NCIB 3610 RM strain. (A) Bacterial cultures were grown to an OD_600_ of 0.5, concentrated and spotted on the plate as described in Materials and Methods and [149]. Each icon represents top-grown image of centrally inoculated Petri plate (diameter 9 cm) containing LB and 0.3% of agar after 5 hours of incubation at 37°C, the white dotted circles denote the boundaries of the swimming discs. The experiment was reproduced three times; results from the representative experiment are presented. (B) Quantitative swimming assay of the NCIB 3610 WT (blue line), NCIB 3610 RM (red line) and NCIB 3610 RM *flgMΔ65* (dotted red line) mutants was performed as in (A) by measurement of swimming discs at indicated time. Values represent the mean of three experiments including two replicas for each strain.
(TIF)

S4 Fig Ectopic expression of the *flhO-flhP* operon does not restore biofim formation in *B*. *subtilis* RM cells. Colony and pellicle biofilm formation by *B*. *subtilis* NCIB 3610 WT and RM cells. The colony column shows individual colonies grown on MSgg agar medium for 72h at 30°C. The scale bar is 5mm. The pellicle column shows microtitre wells (diameter 1.5 cm) in which cells were grown in MSgg medium without agitation for 72h at 30°C. The scale bar is 5 mm. The relevant mutant genotypes are indicated on the side of each image. For the colony assay, 2μl of culture was spotted onto MSgg agar plate and, for pellicle assay, was added to 2ml of MSgg medium in a well of 24-well sterile microtiter plate. The experiment was reproduced four times. The results from the representative experiment are presented.
(TIF)

S5 Fig Inactivation of the *abrB* does not restore biofilm formation by *B*. *subtilis* RM and double RM *sinR* mutant strains. Pellicle biofilm formation by *B*. *subtilis* NCIB 3610 *abrB*, NCIB 3610 *sinR*, *abrB* strains and their respective RM derivatives. Relevant genotypes are indicated on the top of each column. The images show microtitre wells (diameter 1.5 cm) in which cells were grown in MSgg medium without agitation for 72h at 30°C. The experiment was reproduced three times including three replicas for each strain. Presented are the results from the representative experiment.
(TIF)

S6 Fig Modification of the phosphorelay in *B*. *subtilis* RM cells. Schematic representation of *the* multicomponent Spo0A phosphorelay. Only the key elements relevant to this study are shown. Phosphoryl groups are transferred from sensor protein kinases (KinA-E) to Spo0F, Spo0B, and ultimately to Spo0A. KbaA protein stimulates KinB activity. SivA and SivB proteins inhibit KinA autophosphorelation. RapA and RapB proteins dephosphorylate Spo0F∼P. PhrA peptide antagonizes Spo0F dephosphorylation by inhibiting RapA. The Spo0E and YisI phosphatases dephosphorylate *B*. *subtilis* Spo0A. Sporulation is triggered when the level of Spo0A∼P reaches a high threshold level. Orange, blue and uncolored rectangles indicate the phosphorelay components which are, respectively, up-regulated, down-regulated and unaffected in the RM cells (categorized according to joint results of transcriptome and proteome analyses). The arrows and the bar-headed lines indicate positive and negative activities, respectively.
(TIF)

S7 Fig Effect of Rho inactivation on sporulation efficiency of the PY79 strain mainly involves KinB. (A) Rho inactivation partially restores sporulation in the *kinA* mutant and has no effect in the *kinB* mutant. Sporulation efficiency of the PY79 WT, PY79 RM strains and their respective *kinA* and *kinB* mutants. Cells were inoculated in DS medium at OD_600_ 0.05, incubated at 37° during 20 hours and analyzed for heat resistant spores contents as in Fig 7. Totally, twelve biological replicas of each strain were analyzed in three independent experiments. Plotted are the average values and the standard deviations established in per-cents. (B) Rho inactivation does not modify sporulation efficiency of the *kinA kinB* double mutant. The PY79 *kinA kinB* double and the PY79 *kinA kinB rho* triple mutants were analyzed for sporulation efficiency together with their *kinA* and *kinA rh*o counterparts as in (A). Average values and standard deviations were multiplied by 100 and plotted in a log10 scale; spore percentages are indicated in bracets. The experiment was performed twice and included eight biological replicas of the *kinA kinB* and *kinA kinB rho* strains and four replicas of the *kinA* and *kinA rh*o strains. The data presented in (B) are independent from (A).
(TIF)

S8 Fig The tagged Rho-SPA protein remains functional. (A and B) Quantitative swarming motility assay of the NCIB 3610 (blue line), NCIB 3610 RM (red line) and NCIB 3610 *rho-spa* (dotted red line) strains. Bacterial cultures were grown to an OD_600_ of 0.5.concentrated and spotted on the plate as described (Materials and Methods). Plates were incubated at 37°C for 20 hours. Each icon represents top-grown image of centrally inoculated Petri plate (diameter 9 cm) containing LB and 0.7% of agar. Relevant genotypes are indicated on the side of each image. The experiment was reproduced four times; results from the representative experiment are presented. Values represent the mean of at least three experiments. (C) Colony biofilm formation by the NCIB 3610, NCIB 3610 RM and NCIB 3610 *rho-SPA* cells. The colony column shows individual colonies grown on MSgg agar medium for 72h at 30°C. The relevant genotypes are indicated on the side of each image. The experiment was reproduced three times. The results from the representative experiment are presented. (D) Kinetics of luciferase expression from *gerE-luc* fusion in the BSB1 WT (blue line), BSB1 RM (red line) and BSB1 *rho-spa* (violet line) cells during growth in sporulation-inducing DS medium as described in Materials and Methods and Fig 6. The experiment was reproduced three times. For each strain, plotted are the mean values of relative luminescence readings corrected for OD from four independent cultures analyzed simultaneously. The results from the representative experiment are presented.
(TIF)

S9 Fig Over-production of Rho-SPA protein in the *B*. *subtilis* WT (pRho-SPA) cells. (A) *B*. *subtilis* BSB1 WT cells containing *rho*-SPA translational fusion in the native chromosomal locus (lines 1 and 3) or cloned at pDG148 plasmid (lines 2 and 4; Materials and Methods) were grown in LB medium at 37°C to OD_600_ ∼ 0.5 and analyzed for Rho-SPA protein as described in Materials and Methods. To control equilibrium between the samples, total protein extracts were analyzed for MreB protein using anti-MreB specific antibodies. (B) The amount of Rho-SPA protein was quantified by ImageLab 5.0 software of ChemiDoc MP System (BioRad) using weakly exposed images of the immunoblots from (A). Plotted are the normalized levels of Rho-SPA expressed from the chromosome (WT::*rho*-SPA) or the plasmid WT(pRho-SPA); the chromosomal expression is taken for 1. Values are means of two independent experiments each including two biological replicas.
(TIF)

S1 Table Differential expression analysis of *B*. *subtilis* 1012 and NCIB 3610 RM vs. WT cells. (XLSX)

S2 Table Functional enrichment analysis of differentially expressed genes. (XLSX)

S3 Table Proteomic raw data. (XLSX)

S4 Table asRNAs specific for the SigD regulon. (XLSX)

S5 Table Transcription levels in the 5'-part, central-part and 3'-part of the *kinB* gene across 275 RNA samples. (XLSX)

S6 Table Strains and plasmids used in this study. (DOCX)

S7 Table Oligonucleotides used for strains construction. (DOCX)
