# Supplementary figures and images for: Termination factor Rho: From the control of pervasive transcription to cell fate determination in Bacillus subtilis
Source: PLoS Genet. 2017 Jul 19;13(7):e1006909. doi: 10.1371/journal.pgen.1006909 (PMC5540618; doi:10.1371/journal.pgen.1006909)

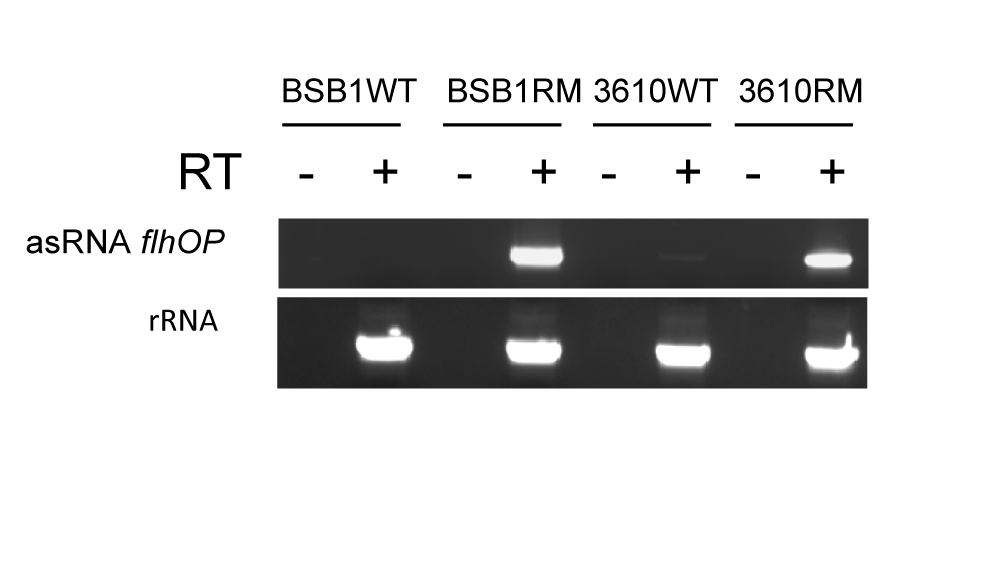

Supplement: S1 Fig — Total RNA was extracted as described in Materials and Methods and cDNA synthesis was performed using 50 ng of total RNA as template and mixture of specific oligonucleotides. Reactions were performed with (+) and without (−) reverse transcriptase (RT). PCR was done with oligonucleotides specific for flhO asRNA (asRNA flhO, top section) and for rRNA (rRNA, bottom section) (S5 Table). (TIF) [file pgen.1006909.s001.tif]

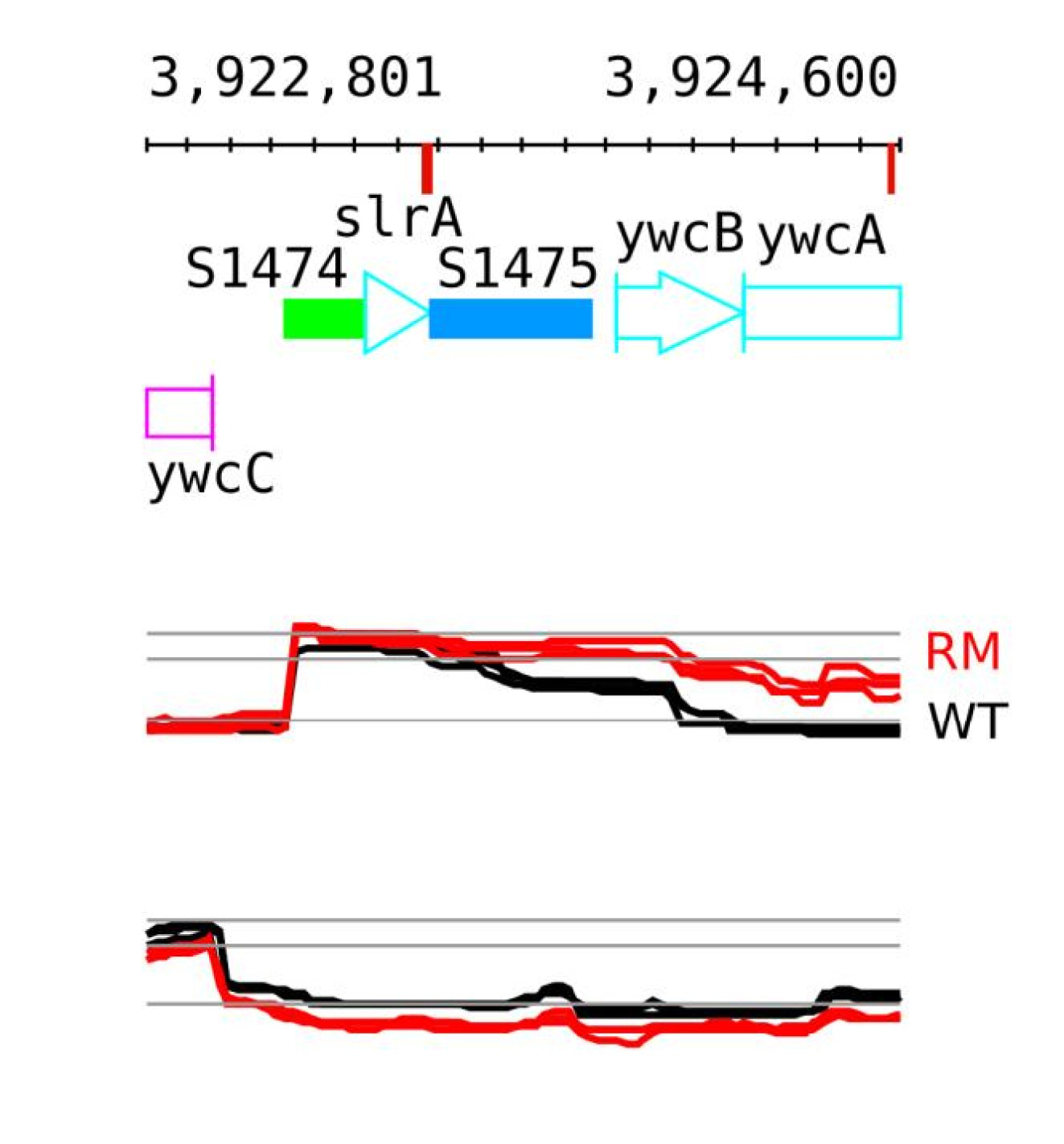

Supplement: S2 Fig — Genomic region corresponding to the slrA gene shows the extension of S1475 RNA (can be visualized on http://genome.jouy.inra.fr/cgi-bin/seb/index.py). Sections show annotated genome (top) and expression profiles of WT (black) and RM (red) on the (+) and (–) strands (mid and bottom sections). (TIF) [file pgen.1006909.s002.tif]

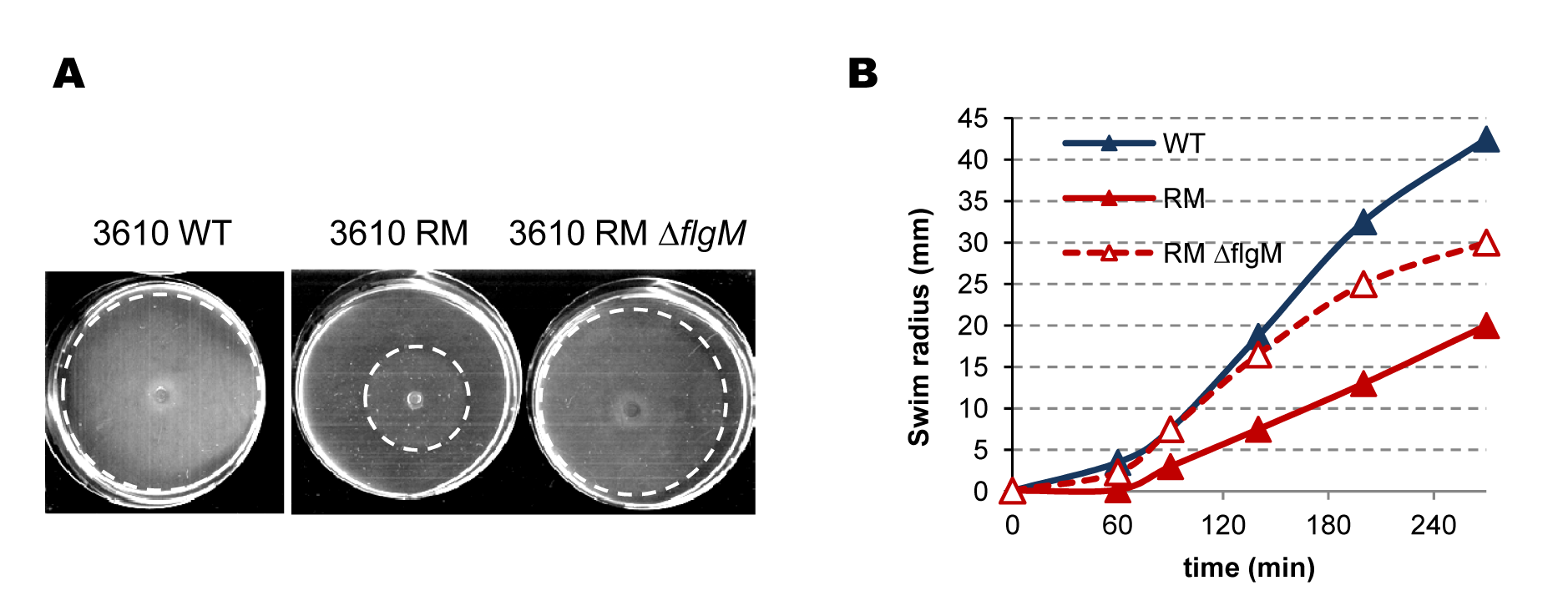

Supplement: S3 Fig — (A) Bacterial cultures were grown to an OD600 of 0.5, concentrated and spotted on the plate as described in Materials and Methods and [149]. Each icon represents top-grown image of centrally inoculated Petri plate (diameter 9 cm) containing LB and 0.3% of agar after 5 hours of incubation at 37°C, the white dotted circles denote the boundaries of the swimming discs. The experiment was reproduced three times; results from the representative experiment are presented. (B) Quantitative swimming assay of the NCIB 3610 WT (blue line), NCIB 3610 RM (red line) and NCIB 3610 RM flgMΔ65 (dotted red line) mutants was performed as in (A) by measurement of swimming discs at indicated time. Values represent the mean of three experiments including two replicas for each strain. (TIF) [file pgen.1006909.s003.tif]

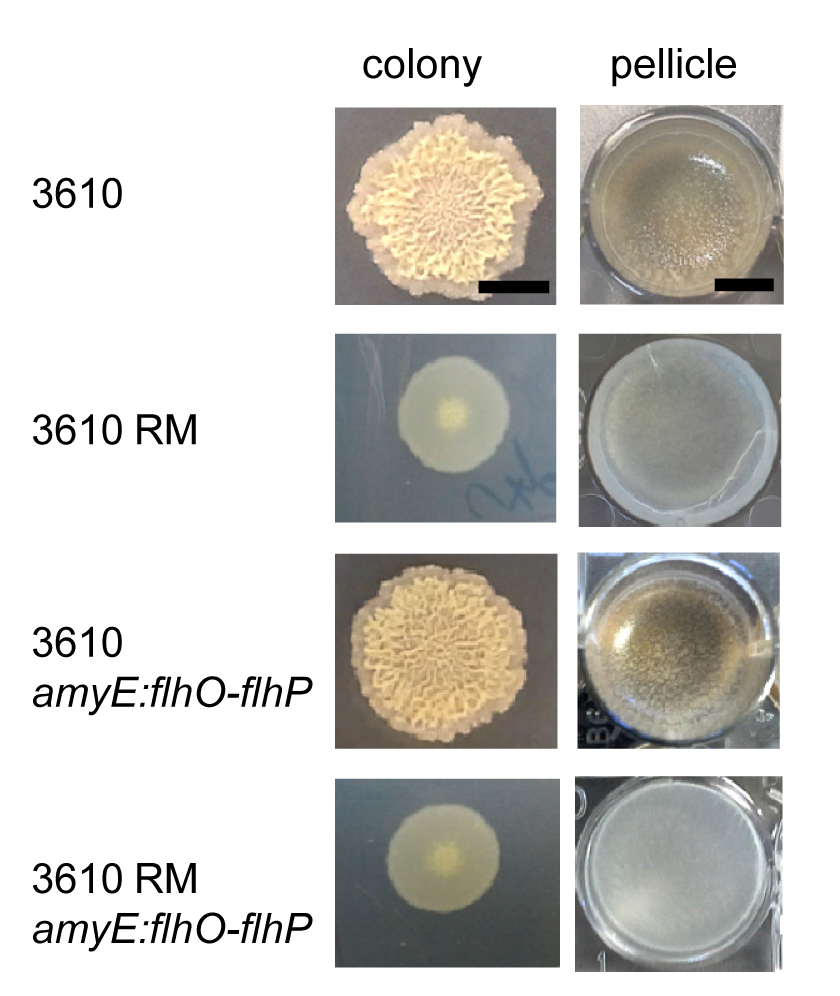

Supplement: S4 Fig — Colony and pellicle biofilm formation by B. subtilis NCIB 3610 WT and RM cells. The colony column shows individual colonies grown on MSgg agar medium for 72h at 30°C. The scale bar is 5mm. The pellicle column shows microtitre wells (diameter 1.5 cm) in which cells were grown in MSgg medium without agitation for 72h at 30°C. The scale bar is 5 mm. The relevant mutant genotypes are indicated on the side of each image. For the colony assay, 2μl of culture was spotted onto MSgg agar plate and, for pellicle assay, was added to 2ml of MSgg medium in a well of 24-well sterile microtiter plate. The experiment was reproduced four times. The results from the representative experiment are presented. (TIF) [file pgen.1006909.s004.tif]

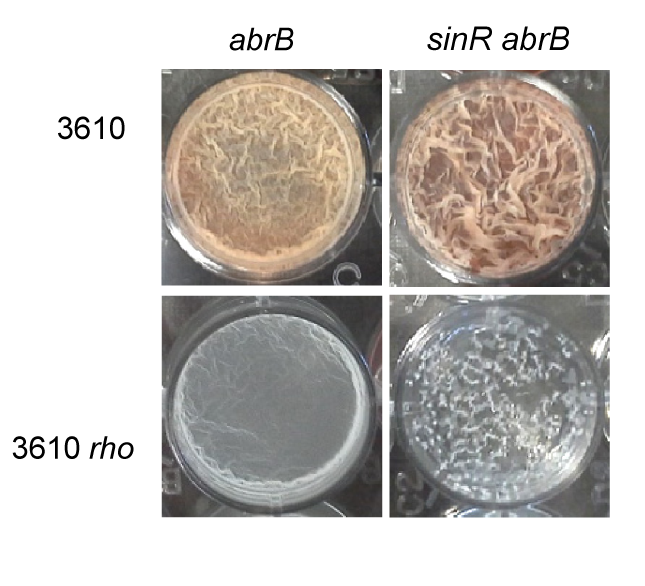

Supplement: S5 Fig — Pellicle biofilm formation by B. subtilis NCIB 3610 abrB, NCIB 3610 sinR, abrB strains and their respective RM derivatives. Relevant genotypes are indicated on the top of each column. The images show microtitre wells (diameter 1.5 cm) in which cells were grown in MSgg medium without agitation for 72h at 30°C. The experiment was reproduced three times including three replicas for each strain. Presented are the results from the representative experiment. (TIF) [file pgen.1006909.s005.tif]

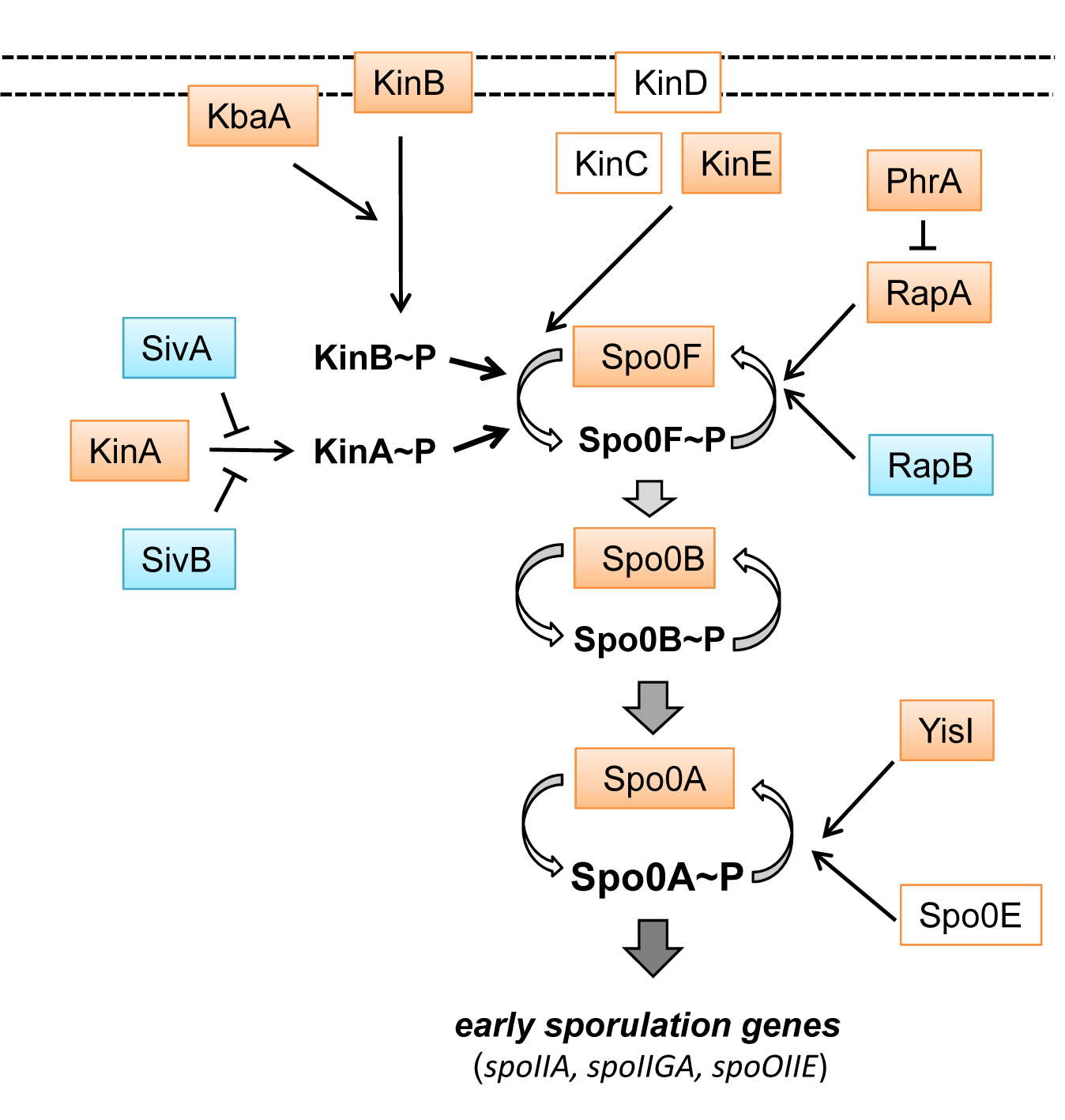

Supplement: S6 Fig — Schematic representation of the multicomponent Spo0A phosphorelay. Only the key elements relevant to this study are shown. Phosphoryl groups are transferred from sensor protein kinases (KinA-E) to Spo0F, Spo0B, and ultimately to Spo0A. KbaA protein stimulates KinB activity. SivA and SivB proteins inhibit KinA autophosphorelation. RapA and RapB proteins dephosphorylate Spo0F∼P. PhrA peptide antagonizes Spo0F dephosphorylation by inhibiting RapA. The Spo0E and YisI phosphatases dephosphorylate B. subtilis Spo0A. Sporulation is triggered when the level of Spo0A∼P reaches a high threshold level. Orange, blue and uncolored rectangles indicate the phosphorelay components which are, respectively, up-regulated, down-regulated and unaffected in the RM cells (categorized according to joint results of transcriptome and proteome analyses). The arrows and the bar-headed lines indicate positive and negative activities, respectively. (TIF) [file pgen.1006909.s006.tif]

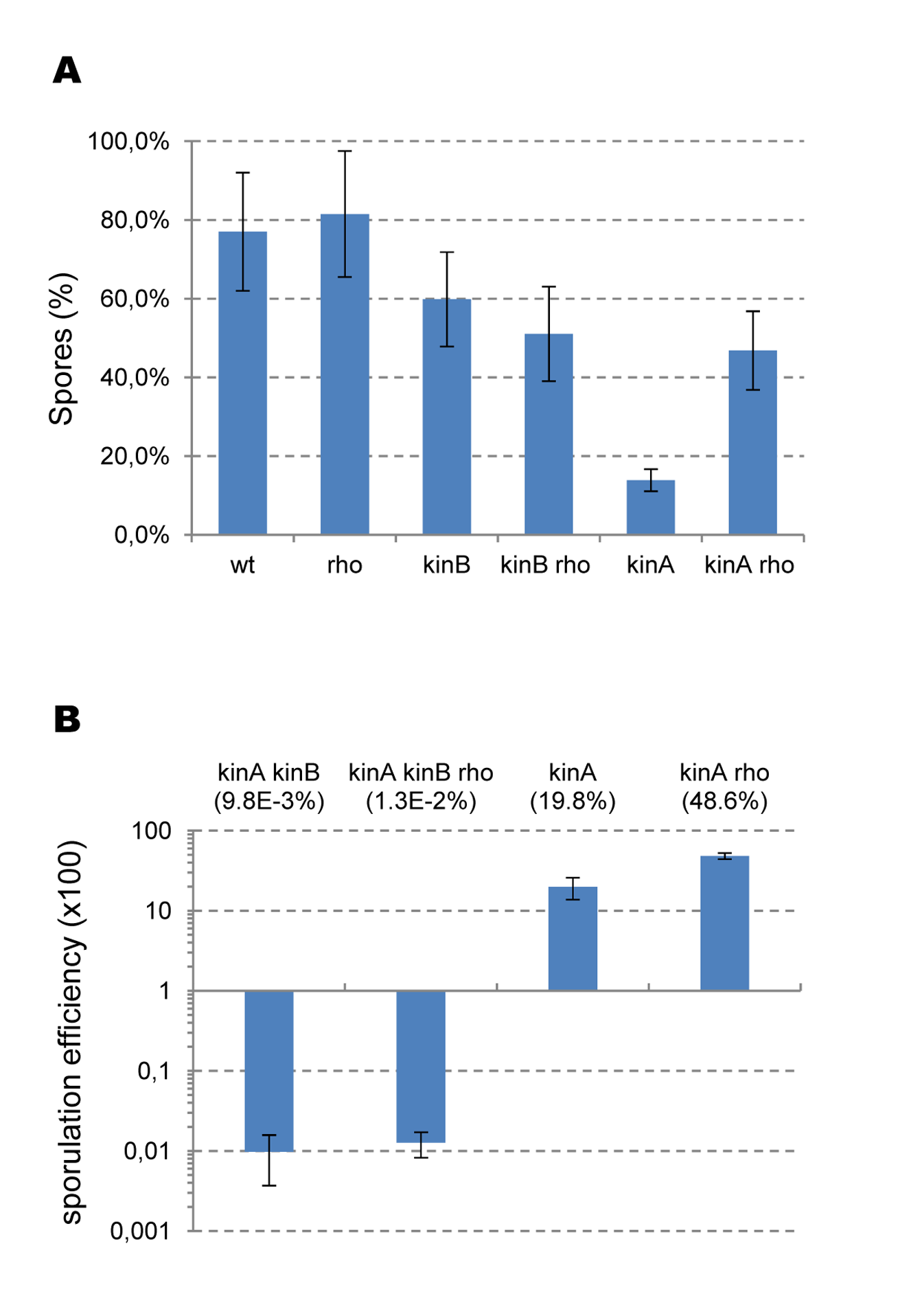

Supplement: S7 Fig — (A) Rho inactivation partially restores sporulation in the kinA mutant and has no effect in the kinB mutant. Sporulation efficiency of the PY79 WT, PY79 RM strains and their respective kinA and kinB mutants. Cells were inoculated in DS medium at OD600 0.05, incubated at 37° during 20 hours and analyzed for heat resistant spores contents as in Fig 7. Totally, twelve biological replicas of each strain were analyzed in three independent experiments. Plotted are the average values and the standard deviations established in per-cents. (B) Rho inactivation does not modify sporulation efficiency of the kinA kinB double mutant. The PY79 kinA kinB double and the PY79 kinA kinB rho triple mutants were analyzed for sporulation efficiency together with their kinA and kinA rho counterparts as in (A). Average values and standard deviations were multiplied by 100 and plotted in a log10 scale; spore percentages are indicated in bracets. The experiment was performed twice and included eight biological replicas of the kinA kinB and kinA kinB rho strains and four replicas of the kinA and kinA rho strains. The data presented in (B) are independent from (A). (TIF) [file pgen.1006909.s007.tif]

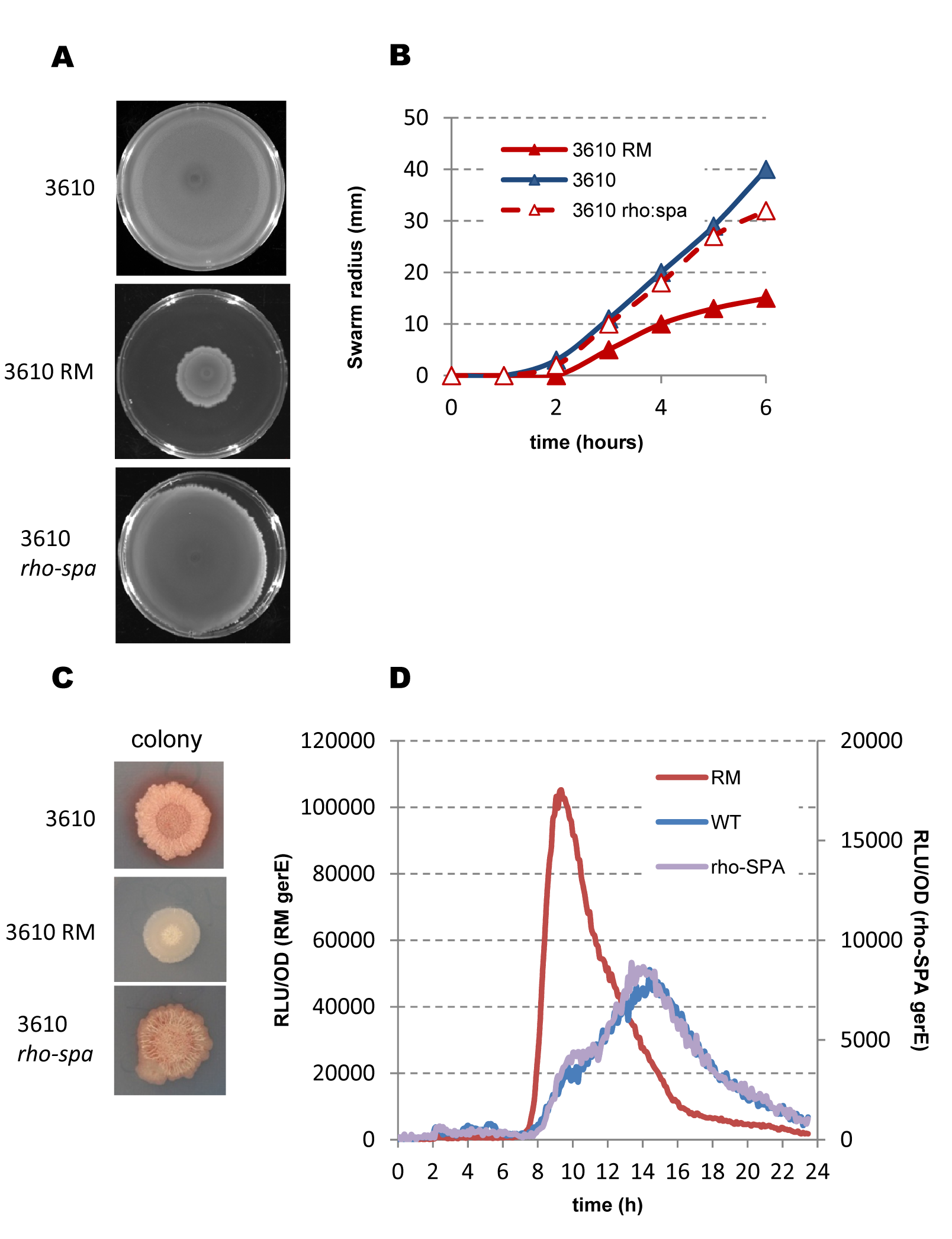

Supplement: S8 Fig — (A and B) Quantitative swarming motility assay of the NCIB 3610 (blue line), NCIB 3610 RM (red line) and NCIB 3610 rho-spa (dotted red line) strains. Bacterial cultures were grown to an OD600 of 0.5.concentrated and spotted on the plate as described (Materials and Methods). Plates were incubated at 37°C for 20 hours. Each icon represents top-grown image of centrally inoculated Petri plate (diameter 9 cm) containing LB and 0.7% of agar. Relevant genotypes are indicated on the side of each image. The experiment was reproduced four times; results from the representative experiment are presented. Values represent the mean of at least three experiments. (C) Colony biofilm formation by the NCIB 3610, NCIB 3610 RM and NCIB 3610 rho-SPA cells. The colony column shows individual colonies grown on MSgg agar medium for 72h at 30°C. The relevant genotypes are indicated on the side of each image. The experiment was reproduced three times. The results from the representative experiment are presented. (D) Kinetics of luciferase expression from gerE-luc fusion in the BSB1 WT (blue line), BSB1 RM (red line) and BSB1 rho-spa (violet line) cells during growth in sporulation-inducing DS medium as described in Materials and Methods and Fig 6. The experiment was reproduced three times. For each strain, plotted are the mean values of relative luminescence readings corrected for OD from four independent cultures analyzed simultaneously. The results from the representative experiment are presented. (TIF) [file pgen.1006909.s008.tif]

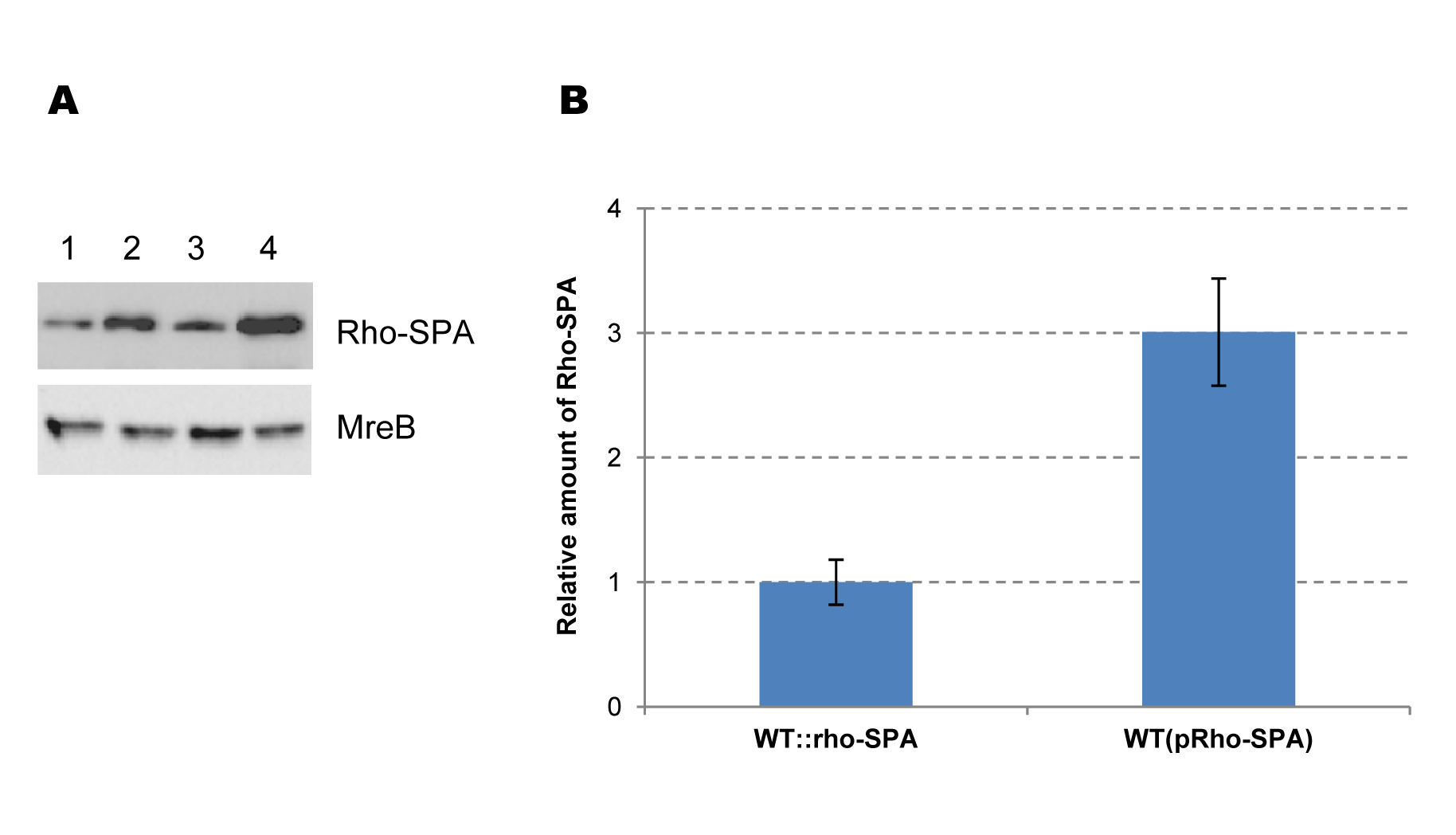

Supplement: S9 Fig — (A) B. subtilis BSB1 WT cells containing rho-SPA translational fusion in the native chromosomal locus (lines 1 and 3) or cloned at pDG148 plasmid (lines 2 and 4; Materials and Methods) were grown in LB medium at 37°C to OD600 ∼ 0.5 and analyzed for Rho-SPA protein as described in Materials and Methods. To control equilibrium between the samples, total protein extracts were analyzed for MreB protein using anti-MreB specific antibodies. (B) The amount of Rho-SPA protein was quantified by ImageLab 5.0 software of ChemiDoc MP System (BioRad) using weakly exposed images of the immunoblots from (A). Plotted are the normalized levels of Rho-SPA expressed from the chromosome (WT::rho-SPA) or the plasmid WT(pRho-SPA); the chromosomal expression is taken for 1. Values are means of two independent experiments each including two biological replicas. (TIF) [file pgen.1006909.s009.tif]
