# Supplementary material for: Termination factor Rho: From the control of pervasive transcription to cell fate determination in Bacillus subtilis
Source: PLoS Genet. 2017 Jul 19;13(7):e1006909. doi: 10.1371/journal.pgen.1006909 (PMC5540618; doi:10.1371/journal.pgen.1006909)
Supplement: S6 Table — (DOCX) [file pgen.1006909.s015.docx]

**S6 Table. Strains and plasmids used in this study.**

| **Strain** | **Genotype** | | **Source or reference** |
| --- | --- | --- | --- |
| 1012 | *B. subtilis 168 leuA8 metB5hsrM1* | | Nicolas et al., 2012 |
| 1012 RM | *1012Δrho::phleo* | | Nicolas et al., 2012 |
| BSB1 | *B. subtilis* 168 *trp^+^* | | Nicolas et al., 2012 |
| PY79 | *B. subtilis* prototroph SPβ-cured | | Youngman et al., 1984 |
| NCIB3610 | *B. subtilis* prototroph | | Zeigler et al., 2008 |
| TF8A | *B. subtilis* 168 trpC2 ΔSPβ Δskin ΔPBSX | | Westers et al., 2003 |
| BRL1 | BSB1 *Δrho::phleo* | | This study |
| BRL152 | PY79 *Δrho::phleo* | | This study |
| BRL149 | 3610 *Δrho::phleo* | | This study |
| BRL146 | TF8A *Δrho::phleo* | | This study |
| BG11858 | *slrR::pMutin (erm^R^)* | | Kobayashi et al., 2003 |
| BRL161 | BSB1 *amyE::P_flhOP_-flhOP* | | This study |
| BRL169 | 3610 *amyE::P_flhOP_-flhOP* | | This study |
| BRL170 | 3610 *Δrho::phleo* *amyE::P_flhOP_-flhOP* | | This study |
| BRL221 | 3610 *slrR::pMutin* | | This study |
| BRL222 | 3610 *Δrho::phleo* *slrR::pMutin* | | This study |
| BRL513 | 3610 *Δrho::phleo* *flgMΔ63* | | This study |
| BRL223 | 3610 *slrR::pMutin amyE::P_flhOP_-flhOP* | | This study |
| BRL224 | 3610 *Δrho::phleo* *slrR::pMutin amyE::P_flhOP_-flhOP* | | This study |
| BRL504 | 3610 *rho* wt^*^ | | This study |
| ABS840 | *ΔsinR::cam* | | A. Chastanet, personal collection |
| BMR50 | *abrB::phleo* | | Kobir et al., 2014 |
| BSB1 RhoSp | BSB1* Δrho::spc* | | BaSysBio consortium collection |
| BRL212 | 3610 *ΔsinR::cam* | | This study |
| BRL334 | 3610 *abrB::phleo* | | This study |
| BRL341 | 3610 *ΔsinR::cat abrB::phleo* | | This study |
| BRL333 | 3610*Δrho::spc* | | This study |
| BRL339 | 3610*Δrho::spc sinR::cam* | | This study |
| BRL340 | 3610*Δrho::spc abrB::phleo* | | This study |
| BRL343 | 3610*Δrho::spc sinR::Cam abrB::phleo* | | This study |
| ABS1081 | PY79 *ΔkinA::tet* | | A. Chastanet, personal collection |
| BRL176 | PY79 *kinB::pMutin (erm^R^)* | | This study |
| BRL236 | PY79 *ΔkinA::tet kinB:: pMutin* | | This study |
| BRL240 | PY79 *Δrho::phleo* *kinA::tet* | | This study |
| BRL177 | PY79 *Δrho::phleo kinB::pMutin* | | This study |
| BRL237 | PY79 *Δrho::phleo* *kinA::tet kinB::pMutin* | | This study |
| BRL154 | BSB1 *kinB::pMutin (erm^R^)* | | This study |
| BRL565 | BSB1 *Δrho::phleo kinB::pMutin* | | This study |
| BRL306 | BSB1 *ΔkinA::tet* | | This study |
| BRL307 | BSB1 *Δrho::phleo* *kinA::tet* | |  |
| PP530 | *spo0A-luc* | | Mirouze et al., 2011 |
| BRL116 | BSB1 *spo0A-luc* | | This study |
| BRL122 | BSB1* Δrho::phleo spo0A-luc* | | This study |
| BRL111 | BSB1 *spoIIA-luc* | | This study |
| BRL117 | BSB1 *Δrho::phleo spoIIA-luc* | | This study |
| BRL114 | BSB1 *gerE-luc* | | This study |
| BRL120 | BSB1 *Δrho::phleo gerE-luc* | | This study |
| BRL327 | BSB1 *eps-luc* | | This study |
| BRL328 | BSB1 *Δrho::phleo eps-luc* | | This study |
| BRL329 | BSB1 *tapA-luc* | | This study |
| BRL330 | BSB1*Δrho::phleo tapA-luc* | | This study |
| BRL266 | BSB1 *spoIIA-luc kinA::tet* | | This study |
| BRL267 | BSB1 *spoIIA-luc kinB::pMutin* | | This study |
| BRL566 | BSB1 *Δrho::phleo spoIIA-luc kinA::tet* | | This study |
| BRL567 | BSB1 *Δrho::phleo spoIIA-luc kinB::pMutin* | | This study |
| BRL193 | BSB1 *kinA-SPA* | | This study |
| BRL195 | BSB1 *Δrho::phleo kinA-SPA* | | This study |
| BRL139 | BSB1 *kinB-SPA* | | This study |
| BRL142 | BSB1 *Δrho::phleo kinB-SPA* | | This study |
| BRL415 | BSB1 *rho-SPA* | | This study |
| **Plasmids** | | | |
| pMutin4 | | Erm^R^, Amp^R^ | Vagner et al., 1998 |
| pUC18-Luc | | Cam^R^, Amp^R^ | Mirouze et al., 2011 |
| pSG1729 | | Spc^R^, Amp^R^ | Lewis and Marston, 1999 |
| pMUTIN-SPA | | Erm^R^, Amp^R^ | Lecointe et al., 2007 |
| pMUTINLICSPA | | Erm^R^, Amp^R^ | Doherty et al., 2010 |
| pMAD | | Erm^R^, Amp^R^ | Arnaud et al., 2004 |
| pDG148 | | Kan^R^, Phleo^R^, Amp^R^ | Stragier et al., 1988 |
| p148Δ | | pDG148ΔlacI; Kan^R^, Phleo^R^, Amp^R^ | This study |
| pRho | | P*_spac_-rho* at pDG148ΔlacI; Kan^R^, Phleo^R^, Amp^R^ | This study |
| pRho-SPA | | P*_spac_-rho-SPA* at pDG148ΔlacI; Kan^R^, Phleo^R^, Amp^R^ | This study |
| pGKV210 | | Erm^R^ | van der Vossen *et al*., 1985 |
| pKinB-S | | P_kinB_-*kinB* (157bp) at pGKV210; Erm^R^, Cam^R^, Amp^R^ | This study |
| pKinB-L | | P_kinB_-*kinB* (417 bp) at pGKV210; Erm^R^, Cam^R^, Amp^R^ | This study |
| pKinB-L-RBSm+ | | Modified pKinB-L with a strong *kinB* translation initiation region | This study |
| pKinB-L-RBSm- | | Modified pKinB-L with a weak *kinB* translation initiation region | This study |
| pKinB-S-RBSm+ | | Modified pKinB-L with a strong *kinB* translation initiation region | This study |
| pKinB-S-RBSm- | | Modified pKinB-L with a weak *kinB* translation initiation region | This study |

1. Youngman P, Perkins J, & Losick R. Construction of a cloning site near one end of Tn917 into which foreign DNA may be inserted without affecting transposition in Bacillus subtilis or expression of the transposon-borne erm gene. Plasmid. 1984; 12: 1-9.
2. Zeigler DR, Pràgai Z, Rodriguez S, Chevreux B, Muffler A, Albert T, et al. The origins of BSB1, W23, and other *Bacillus subtilis* legacy strains. J Bacteriol. 2008; 190: 6983-6995.
3. Westers H, Dorenbos R, van Dijl JM, Kabel J, Flanagan T, Devine KM. Genome engineering reveals large dispensable regions in *Bacillus subtilis*. Mol Biol Evol. 2003; 20:2076–2090.
4. Kobayashi K, Ehrlich SD, Albertini A, Amati G, Andersen KK, Arnaud M, et al. Essential *Bacillus subtilis* genes. Proc Natl Acad Sci U S A. 2003; 100: 4678–4683.
5. Kobir A, Poncet S, Bidnenko V, Delumeau O, Jers C, Zouhir S, et al. Phosphorylation of *Bacillus subtilis* gene regulator AbrB modulates its DNA-binding properties. Mol Microbiol. 2014; 92:1129-1141.
6. Vagner V, Dervin E, & Ehrlich SD. A vector for systematic gene inactivation in *Bacillus subtilis*. Microbiology. 1998; 144: 3097-3104.
7. Lewis PJ, & Marston AL. GFP vectors for controlled expression and dual labeling of protein fusions in *Bacillus subtilis*. Gene. 1999; 227: 101-109.
8. Lecointe F, Serena C, Velten M, Costes A, McGovern S, Meile J-C, et al. Anticipating chromosomal replication fork arrest: SSB targets repair DNA helicases to active forks. The EMBO Journal. 2007; 26: 4239-4251.
9. Doherty GP, Fogg MJ, Wilkinson AJ, and Lewis PJ. Small subunits of RNA polymerase: localization, levels and implications for core enzyme composition. Microbiology. 2010; 156: 3532-3543.
10. Stragier P, Bonamy C, and Karmazyn-Campelli C. Processing of a sporulation sigma factor in *Bacillus subtilis*: how morphological structure could control gene expression. Cell. 1988; 52: 697-704.
11. van der Vossen JMBM, Kok J, and Venema J. Construction of cloning, promoter-screening, and terminator-screening shuttle vectors for *Bacillus subtilis* and *Streptococcus lactis*. Appl. Environ. Microbiol. 1985; 50: 540-542.
12. Arnaud M, Chastanet A, Débarbouillé M. New vector for efficient allelic replacement in naturally nontransformable, low-GC-content, Gram-positive bacteria. App Environ Microbiol. 2004; 70: 6887-6891.
