# Supplementary material for: Termination factor Rho: From the control of pervasive transcription to cell fate determination in Bacillus subtilis
Source: PLoS Genet. 2017 Jul 19;13(7):e1006909. doi: 10.1371/journal.pgen.1006909 (PMC5540618; doi:10.1371/journal.pgen.1006909)
Supplement: S7 Table — (DOCX) [file pgen.1006909.s016.docx]

**S7 Table. Oligonucleotides used for strains construction**

| Oligonucleotide | Sequence (5’-> 3’) |
| --- | --- |
| eb423 | GCGGCTAGCGAAAAGCGTGGTGTTTTTATG |
| eb424 | GCGGATCCTTACCTTCTTGCAGATGATAG |
| eb460 | GCCAAGCTTCTTAATCAGGGAGGCTTTTTG |
| eb461 | CGGGATCCGATCAATGTAACGTTTGTTTTGAT |
| eb617 | AGGGTACCTTCACGAAAAGATGTTAAATG |
| eb618 | CGGGATCCCATTTTCTTATTATCTGAC |
| eb625 | ATGGGATCCGCAACCCTCTTACAG |
| eb626 | CTAGCCATGGTTTTTTTGGAAATGAAATTTTAAACG |
| veb606 | GACGGTACCCTACAAAACAAGTCG |
| veb607 | CTACCATGGGTGAGGAAGATCAGCG |
| veb608 | TAAAAGCTTTATCTTCTGCACATCTG |
| veb610 | AAGAATTCAAGGCGGAAAGAATC |
| veb611 | ACAAAGCTTAGTGACCCAAG |
| gerE-fwd | GATGAAGCTTCCCGCTTATATTG |
| gerE-rev | ATGAAGCTTCCGTCGCCATGGCTG |
| spo2A-fwd | ATGAAGCTTCCGTCGCCATGGCTG |
| spo2A-rev | TATGGATCCGGATAATGAGTGTTTCGATTTCG |
| F-pUC18-luc | CTCTAGAGGATCCCCGGGTACCAG |
| R-pUC18-luc | TCGACCTGCAGGCATGCAAGCTTG |
| epsA-fwd* | **CAAGCTTGCATGCCTGCAGGTCGA**GACGGCTGCGGGCAAATAGAGCC |
| epsA-rev* | **CTGGTACCCGGGGATCCTCTAGAG**TCCCGCGGCTGGCTTCCCGCG |
| tapA-fwd* | **CAAGCTTGCATGCCTGCAGGTCGA**CAACGGATTCGGGAACAGAAAG |
| tapA-rev* | **CTGGTACCCGGGGATCCTCTAGAG**CTGTAAAACACTGTAACTTGATATGACAAT |
| rho-SPA-fwd** | GGGTTCCTGGCGCGAGCGGAAGAACGCTTTCCGGGGGG |
| rho-SPA-rev** | TTGGGCTGGCGCGAGCCCTTCTTGCAGATGATAGATT |
| eb700 | CATCCGCAATGATTGCCCAGCAGCG |
| eb706 | GTGAAGCGCAATGATCCCGCTCGTC |
| eb717 | CACCCCAATCATCTGTCC CACCTTCGG |
| eb705 | CGCGTTCCCTGAGATGCT |
| eb702 | CATTACCGTCCGCCGTGCTG |
| eb708 | GTCGGATGCGGTTTTTACAAG |
| eb710 | CGGCGATGGTGAACAGAAT |
| eb715 | TCACGRCACGAGCTGACGAC |
| eb716 | CTCCTACGGGAGGCAGC |
| eb406 | GTCACCGAATTGATGGCTTGTC |
| veb676 | TTTGTGAATCGAATTCGAGC |
| veb678 | AGGAGGACAAAC***ATGGAAATTCTAAAAGACTATCTTCTGC*** |
| veb679 | TATAAAATATGAATCTATTATAACACTAAATATTAG |
| veb690 | TGATAAA***ATTTCACACGAATGGAAATTCTAAAAG*** |
| veb686 | CAAGGATC*C*GAGACAGTTTGC |
| veb687*** | *CCGTTGATTTTCATAGGATTC*CTC |
| veb688*** | *GAATCCTATGAAAATCAACGG*GTCATACAAAGTAGACGCAAATC |
| veb689 | CGAGGTCGACCGGCTCTACTTGTG |

* Bolded are sequences complementary to the oligonucleotides F-pUC18-luc or R-pUC18-luc

** Sequences homologous to *rho* are underlined

*** In italics and underlined are complementary sequences
